# Stepwise Assembly of the *Bacteroides fragilis* Capsular Polysaccharide A Repeating Unit in *Escherichia coli*

**DOI:** 10.1021/acs.biochem.6c00056

**Published:** 2026-03-25

**Authors:** Beth A. Scarbrough, Claire E. Moneghan, Sara Salamat, Manoj K. Dooda, Alexis H. Murray, Jenna S. Costelloe, Matthew A. Jorgenson, Jerry M. Troutman

**Affiliations:** † Department of Chemistry, 14727 University of North Carolina at Charlotte, 9201 University City Blvd, Charlotte, North Carolina 28223, United States; ‡ Department of Biological Sciences, University of North Carolina at Charlotte, 9201 University City Blvd, Charlotte, North Carolina 28223, United States; § Department of Microbiology and Immunology, 12215 University of Arkansas for Medical Sciences, 4301 W. Markham St, Little Rock, Arkansas 72205, United States

## Abstract

Bacterial surface polysaccharides are versatile structures that provide specificity to the behavior and interactions of a given bacterial strain. One surface polysaccharide displayed on the organism *Bacteroides fragilis*, Capsular Polysaccharide A, has been implicated as a potential therapeutic for autoimmune disorders. This polymer is composed of repeating units of the tetrasaccharide 2-acetamido-4-amino-2,4,6-trideoxygalactopyranose (AATGal), 4,6-*O*-pyruvate-galactopyranose (PyrGal), *N*-acetylgalactosamine (GalNAc), and galactofuranose (Gal*f*). While this and other bacterial surface polysaccharides are attractive to study and apply to biomedicine, it can be difficult to acquire quick, inexpensive access to these pure materials. In this work, we developed a recombinant expression system in *Escherichia coli* for the stepwise production of the CPSA polymer. A series of sequential plasmids were prepared, each incorporating successive genes required for CPSA biosynthesis. Using these iterative plasmids, we were able to observe production of the CPSA repeating unit and precursors by liquid chromatography mass spectrometry (LC-MS) analysis of cell lysates. We found that it was critical to include the CPSA polymerase but not the flippase, indicating that a native *E. coli* flippase could support polymer production. We also provide evidence that the CPSA polymer produced by *E. coli* can be ligated to LPS by the *E. coli* WaaL ligase, and deletion of this gene led to the formation of a water-soluble polymer. Overall, this work describes the first recombinant system for CPSA production and outlines a key strategy for the production of complex glycopolymers.

## Graphical abstract

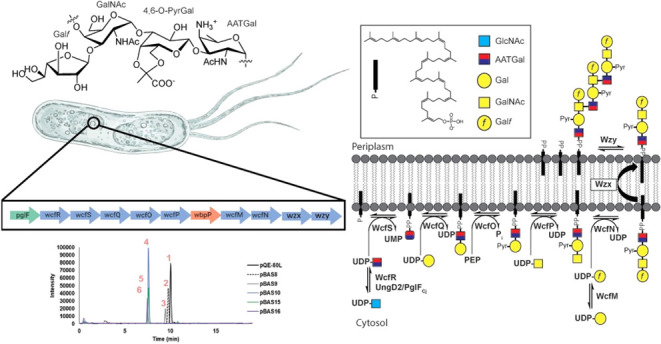

## Introduction

*Bacteroides fragilis* is one of a broad range of microbes associated with the mammalian gut microbiome.^1,2^ It is a Gram-negative obligate anaerobe that is thought to play a central role in host immune system development.^3^ The role of *B. fragilis* in immune system modulation is dependent on its ability to produce the surface polymer capsular polysaccharide A (CPSA) (Figure 1). CPSA belongs to a unique class of zwitterionic polysaccharides that, unlike the more common neutral and negatively charged glycans, can elicit a potent, T-cell dependent immune response.^4,5^ CPSA also activates anti-inflammatory innate immune responses by stimulating the production of anti-inflammatory cytokines, such as IL-10.^6,7^ This has led to CPSA being considered as a potential therapeutic for inflammation-associated diseases such as multiple sclerosis, ulcerative colitis, viral encephalitis, and irritable bowel syndrome.^7–10^

**Figure 1:**
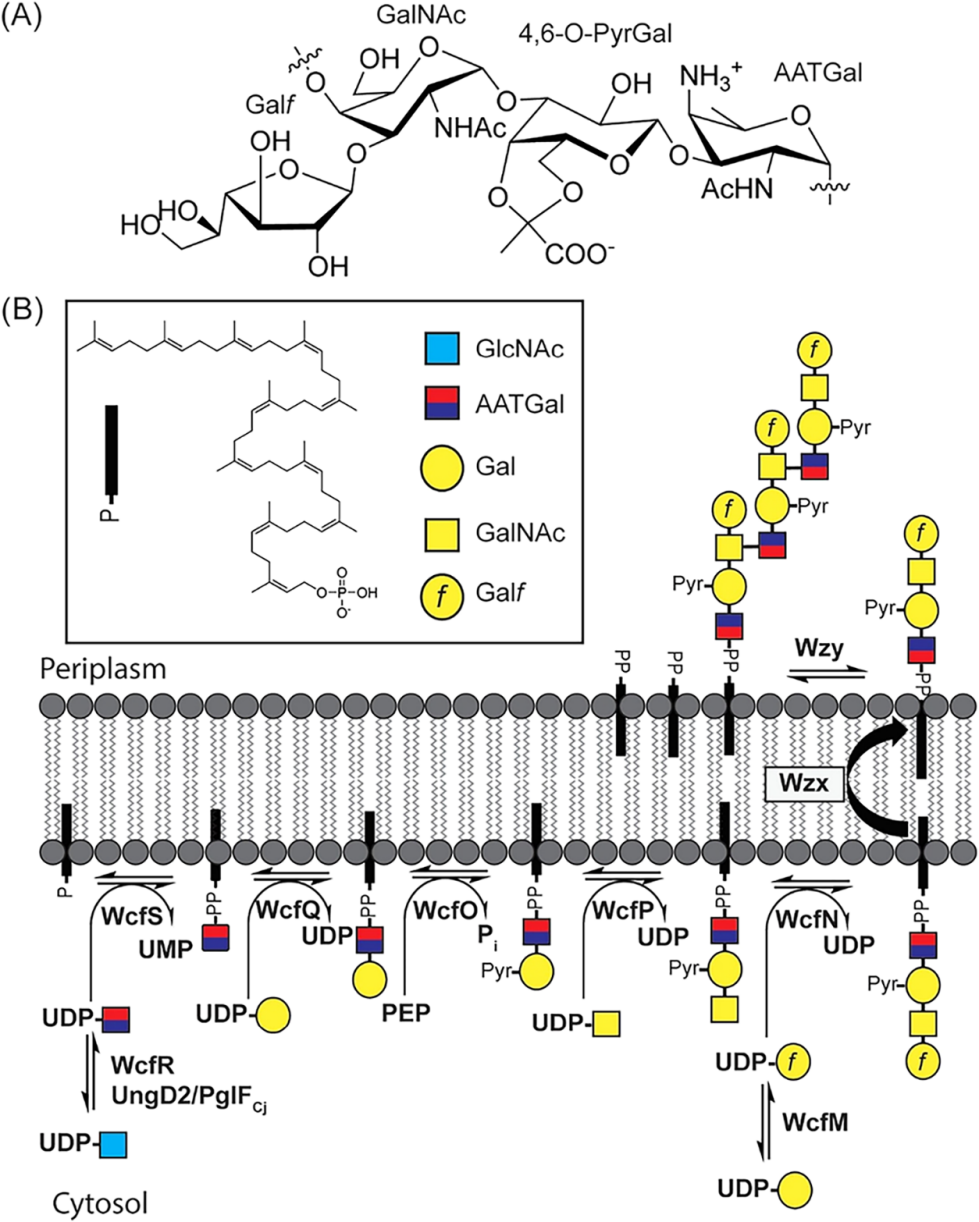
Capsular polysaccharide A. (A) Repeating unit of *B. fragilis* Capsular Polysaccharide A. Wavy lines represent linkage points between repeat units. (B) Biosynthesis pathway for CPSA including sugar modifying enzymes UngD2 from *B. fragilis* and the alternative enzyme PglF from *Campylobacter jejuni* used in this work. Briefly: UDP-GlcNAc is converted to UDP-AATGal through the action of UngD2/PglF and WcfR. The AATGal-P is then transferred to BP by the initiating phosphoglycosyltransferase WcfS. The glycosyltransferase WcfQ then transfers galactose to AATGal and the disaccharide is pyruvylated by WcfO. The pyruvate group is donated from phosphoenolpyruvate (PEP). After pyruvylation WcfP transfers GalNAc and WcfN transfers a galactofuranose formed by WcfM which isomerizes UDP-galactopyranose. The completed repeat unit is then flipped to the periplasmic face of the inner membrane by the flippase Wzx and is polymerized by Wzy prior to transport to the cell surface.

Access to the CPSA polymer is critical for further development as a potential therapeutic. However, isolation of CPSA from *B. fragilis* can be difficult, due to the presence of numerous other capsular sugars on the *B. fragilis* surface, complex growth requirements, and laborious isolation procedures.^11^ In addition, synthetic schemes to prepare CPSA require as many as 20 chemical manipulations to build the repeating unit of the polymer, and several of these repeat units must be linked to provide a biologically active molecule.^12–16^ An alternative to chemical synthesis and isolation from native organisms is the development of methods for the recombinant production of the polymer. The advantage of such systems is the ability to use easily manipulated hosts to prepare the capsule and control the expression of key genes involved in its biosynthesis.^17–20^

To design a recombinant system for polysaccharide production, it is important to understand the biosynthetic pathway that is responsible for its production in the native organism. Previous work has identified the specific roles of genes involved in CPSA production.^21,22^ The CPSA repeating tetrasaccharide consists of 2-acetamido-4-amino-2,4,6-trideoxygalactopyranose (AATGal), 4,6-*O*-pyruvate-galactopyranose (PyrGal), *N*-acetylgalactosamine (GalNAc), and galactofuranose (Gal*f*) (Figure 1).^23^ The polymer is formed in a Wzx/Wzy pathway where it is assembled one sugar at a time on the lipid anchor, bactoprenyl phosphate (BP; also known as undecaprenyl phosphate or Und-P), on the cytoplasmic face of the inner membrane. CPSA biosynthesis begins with the addition of phospho-AATGal to BP by the initiating phosphoglycosyltransferase WcfS to produce BPP-AATGal (Figure 1).^23,24^ The remaining sugars, galactose (Gal), *N*-acetylgalactosamine (GalNAc), and galactofuranose (Gal*f*) are sequentially appended to BPP-AATGal by the glycosyltransferases WcfQ, WcfP, and WcfN, respectively.^21^ In this work, we devised a recombinant system for CPSA production in *E. coli* by the stepwise construction of plasmids that encode the key genes required for the biosynthesis of each intermediate isoprenoid-linked glycan. Incorporation of *B. fragilis* *wzx*/*wzy* genes resulted in the production of a CPSA polymer. We also found that the polymer was formed as either soluble or lipid-A-linked material, and we could control the destination of the glycan through deletion of a ligase involved in conjugating glycans to lipid A.^25–27^ Overall, this work represents the first recombinant production system for potentially therapeutic CPSA.

## Materials and Methods

### General

All bacterial cultures were grown in Miller Lysogeny Broth (Fisher) unless otherwise specified. Liquid chromatography mass spectrometry (LC-MS) analysis was performed on an Agilent 1260 Infinity II instrument equipped with a G6125B single quadrupole mass selective detector. All mobile phases were prepared from LC-MS grade reagents. Immunological detection of CPSA polymer utilized a polyclonal rabbit antibody serum against CPSA that is precleared by extracting nonspecific antibodies with *B. fragilis* that does not produce CPSA.^22,28^

### Construction of CPSA Plasmids

CPSA plasmids were constructed using the primers listed in Supporting Table S1. Briefly, the pQE-80L vector was isolated from *E. coli* DH5α. The vector was digested with restriction enzymes *Bam*HI and *Hin*dIII and purified by gel electrophoresis. Gene inserts were amplified with Phusion DNA polymerase (New England Biolabs). Amplicons were purified using the Wizard SV Gel and PCR Clean-Up System (Promega). Purified, digested vector and inserts were incubated with NEBuilder HiFi DNA Assembly Mix (New England Biolabs) according to the manufacturer’s instructions and incubated at 50 °C, or overnight for constructs with more than four fragments. *E. coli* DH5α cells were then chemically transformed with 5 μL of the assembly reaction mixture and plated on selective media (LB/Carb^100^). Successful transformants were confirmed by colony PCR.

An artificial SacI site was incorporated into pBAS8 immediately following the *wcfS*. For construction of pBAS9–19, SacI digested pBAS8 was used as the vector backbone. For the construction of pBAS16, *wcfO* and *wcfQ* were amplified as a single insert using pBAS10 as a template. For the construction of pBAS 17–19, six genes (*wcfOQP*_Bf_, *wbpP*_Vv_, *wcfMN*) were amplified as a single fragment using pBAS16 as a template. The pBAS17 plasmid full sequence is provided in the Supporting Information (Supporting Figure S1).

### Extraction of BPP-Linked CPSA Intermediates

Cultures for glycolipid extraction were prepared by inoculating a single colony into 5 mL of LB/Carb^100^ and 2% glucose. Cultures were grown overnight for 16 h at 37 °C and 220 rpm. Cell cultures were then diluted 1:1000 into 5 mL of LB/Carb^100^ and grown at 37 °C, 220 rpm until reaching an OD_600_ of approximately 0.6 before induction with 0.1 mM IPTG. After overnight induction, liquid cultures were transferred to falcon tubes and centrifugated at 5000*g* for 15 min at 4 °C. The supernatant was discarded, and the cell pellet was resuspended in 10 mM phosphate-buffered saline (PBS). The cell suspension was then transferred to a 15 mL glass centrifuge tube. To the cell suspension were added 1 mL of chloroform and 2 mL of methanol to create a single-phase solution of water:chloroform:methanol (0.8:1:2). Each sample was vortexed and incubated at room temperature for 20 min to ensure cell lysis. Resulting insoluble materials were clarified from the cell lysate solution by centrifugation at 2500*g* for 20 min at room temperature. The supernatant was then transferred to a clean glass culture tube, frozen at −80 °C, and then dried under vacuum. Dried samples were resuspended in a solution of 1 mM ammonium hydroxide:*n*-propanol (1:1).

### LC-MS Analysis of BPP-Linked CPSA Intermediates

To evaluate the presence of CPSA intermediates in *E. coli*, 10 μL of cell extract was injected and separated using a Waters XBridge BEH C18 column (5 μm, 4.6 mm × 100 mm, 300 Å). For LC-MS analysis, mobile phase A consisted of 0.1% ammonium hydroxide, and mobile phase B was *n*-propanol. BPP-linked intermediates were separated using a 5 – 75% gradient of mobile phase B over 15 min, then 75 – 95% B for 1 min, and an isocratic hold at 95% B for 5 min at 1 mL/min for a total run time of 21 min. A 3 s needle wash was performed between injections. The column was equilibrated for 4 min at 5% B prior to the next injection. Intermediates were detected using SIM of the predicted [M-H]^−1^ and/or [M-H]^−2^ ions of each intermediate. Total ion chromatograms were collected for each injection. Controls were prepared from parent strains expressing empty plasmids and evaluated for CPSA intermediates or isobaric compounds (Supporting Figure S2).

### Cell Fractionation and SDS PAGE

To evaluate CPSA glycoforms in *E. coli*, overnight cultures (5 mL) were prepared from single colonies, induced with 0.1 mM IPTG after reaching an OD_600_ of 0.8_,_ and incubated overnight at 37 °C with shaking at 220 rpm. Cell were pelleted at 5000*g*, resuspended in 0.8 mL of PBS, and transferred to a glass culture tube. To the cell suspensions, 3 mL of a 1:2 solution of chloroform:methanol was added, and the mixture was incubated at room temperature for 20 min. Insoluble material containing LPS was removed from cell suspensions by centrifugation at 2500*g* for 20 min.^29^ The insoluble fraction was washed with a second, single-phase solution of water:chloroform:methanol (0.8:1:2) and dried under vacuum to remove any remaining solvent. The soluble fraction was converted to a two-phase Bligh and Dyer solution by the addition of water and chloroform.^30^ The aqueous and organic phases were separated into two glass tubes and then dried under a vacuum. Dried samples were resuspended in PBS and evaluated by using 10% sodium dodecyl sulfate–polyacrylamide gel electrophoresis (SDS–PAGE) and anti-CPSA blotting. Blots were prepared by transfer of SDS-PAGE separated material to nitrocellulose. The nitrocellulose was blocked with a 5% milk solution for 30 min. The nitrocellulose was then rinsed gently with deionized water (x3) and incubated at room temperature for 1 h with 1 mL of an adsorbed CPSA antiserum diluted 1:30 in sterile 10 mM PBS.^22^ After incubation with anti-CPSA serum, the nitrocellulose was washed twice for 5 min in 0.3% PBST, then placed in alkaline phosphatase conjugated antirabbit goat secondary antibody (1:10,000) for 1 h at room temperature. The nitrocellulose was then washed three times for 1 min with 0.3% PBST and developed with NBT-BCIP.

### SDS-PAGE and LPS Staining

To evaluate LPS and CPS profiles of *E. coli* harboring pBAS16–19, overnight cultures were diluted 1:100 in LB and incubated at 37 °C with shaking until reaching an OD of ∼0.6. Cell cultures were then induced with 0.01 mM IPTG and incubated overnight at 37 °C. Cell cultures were normalized to an OD_600_ of 1.0, and a 0.5 mL volume of each sample was pelleted and resuspended in 100 μL of 1× SDS dye. Cell samples were then lysed at 95 °C for 10 min and cooled to room temperature before 20 μg of Pronase (Sigma) was added. The samples were incubated at room temperature for 20 min prior to 14% SDS-PAGE separation. A 5 μL sample of each lysate was separated at 40 mA for 60 min. Gels were then stained with the Pro-Q Emerald 300 kit (Thermo Fisher Scientific) according to the manufacturer’s instructions. The protein standard was CandyCane Glycoprotein Molecular Weight Standard (Thermo Fisher Scientific).

### Generation of *E. coli* Mutants

*Escherichia coli* mutants MG1655 Δ*wzxB*:frt (BAS5), MG1655 Δ*waaL*:frt (MAJ975), and MG1655 Δ*wza*:frtΔ*waaL*:cam (BAS24) (Supporting Table S2) were prepared using the lambda red recombinase method of Datsenko and Wanner.^31^ Briefly, parent strains of *E. coli* were transformed with pKD46. Single colonies of transformants were used to inoculate 5 mL cultures containing LB/Carb^100^ and incubated at 30 °C. Cultures were induced with 100 mM l-arabinose and returned to the incubator for 1 h at 30 °C. Cells were then pelleted at 5000*g* at 4 °C and washed six times with ice-cold 10% glycerol. These cells were used immediately for transformation or stored as aliquots at −80 °C. For transformation, approximately 1–2 μg of purified amplicon was added to the cell suspensions. Cells were then electroporated at 2500 V and immediately recovered in LB at 30 °C overnight. Recovered cells were plated on LB/Cam^20^ or LB/Kan^50^ to select for mutants. Primers used to generate mutants are listed in Supporting Table S3. Kan resistance cassettes were removed by transforming with pCP20.^32^ To ensure that the Kan resistance cassette was successfully removed, cells were tested for sensitivity against Kan^50^. To cure the cells of the pCP20 plasmid, cells were cultured on LB agar and incubated at 37 °C and the colony purified twice more at 37 °C. Plasmid curing was confirmed by testing cells for sensitivity to Carb^100^.

## Results

### Plasmid Assembly for the Formation of a BPP-Linked AATGal

The first step in CPSA biosynthesis is the addition of AATGal-P to BP to form BPP-AATGal (Figure 2). The AATGal sugar is formed through the oxidation, dehydration, and reduction of UDP-GlcNAc to form UDP-2-acetamido-2,6-dideoxy-α-d-xylo-hexos-4-ulose followed by an amino transfer reaction catalyzed by WcfR to form UDP-AATGal (Figure 2a).^24^ Previous work had identified the gene *ungD2* to encode the likely dehydrogenase that forms the 4-keto sugar substrate for WcfR.^33^ However, this gene was not encoded in the CPSA biosynthesis operon, and previous attempts to overexpress it in *E. coli* were unsuccessful.^24^ The *C. jejuni* gene *pglF* has been readily overexpressed in *E. coli* and serves the same function as the expected product of the *ungD2* gene.^34^ Using the Gibson assembly, we combined *pglF*, *wcfR*, and *wcfS* into plasmid pQE-80L (Table 1, pBAS8). We chose this plasmid due to the presence of a T5 RNA polymerase promoter that could be used with a wide range of *E. coli* strains. We also incorporated a strong *E. coli* ribosome binding site (AGGAGA) before each gene. The plasmid was transformed and expressed in *E. coli* DH5α, and cell lysate extracts were analyzed by LC-MS in selected ion monitoring (SIM) mode for the BPP-linked monosaccharide product of WcfS (Figure 2b). We found that BPP-AATGal was not detected with the empty plasmid (Supporting Figure S2). With the *pglF*, *wcfR*, and *wcfS* genes inserted, a clear BPP-AATGal peak was observed with an *m*/*z* of 1111.5 consistent with the [M-H]^−1^ species and the mass spectrum corresponded to the isoprenoid-linked monosaccharide mass as the major component (Figure 2c).

**Figure 2:**
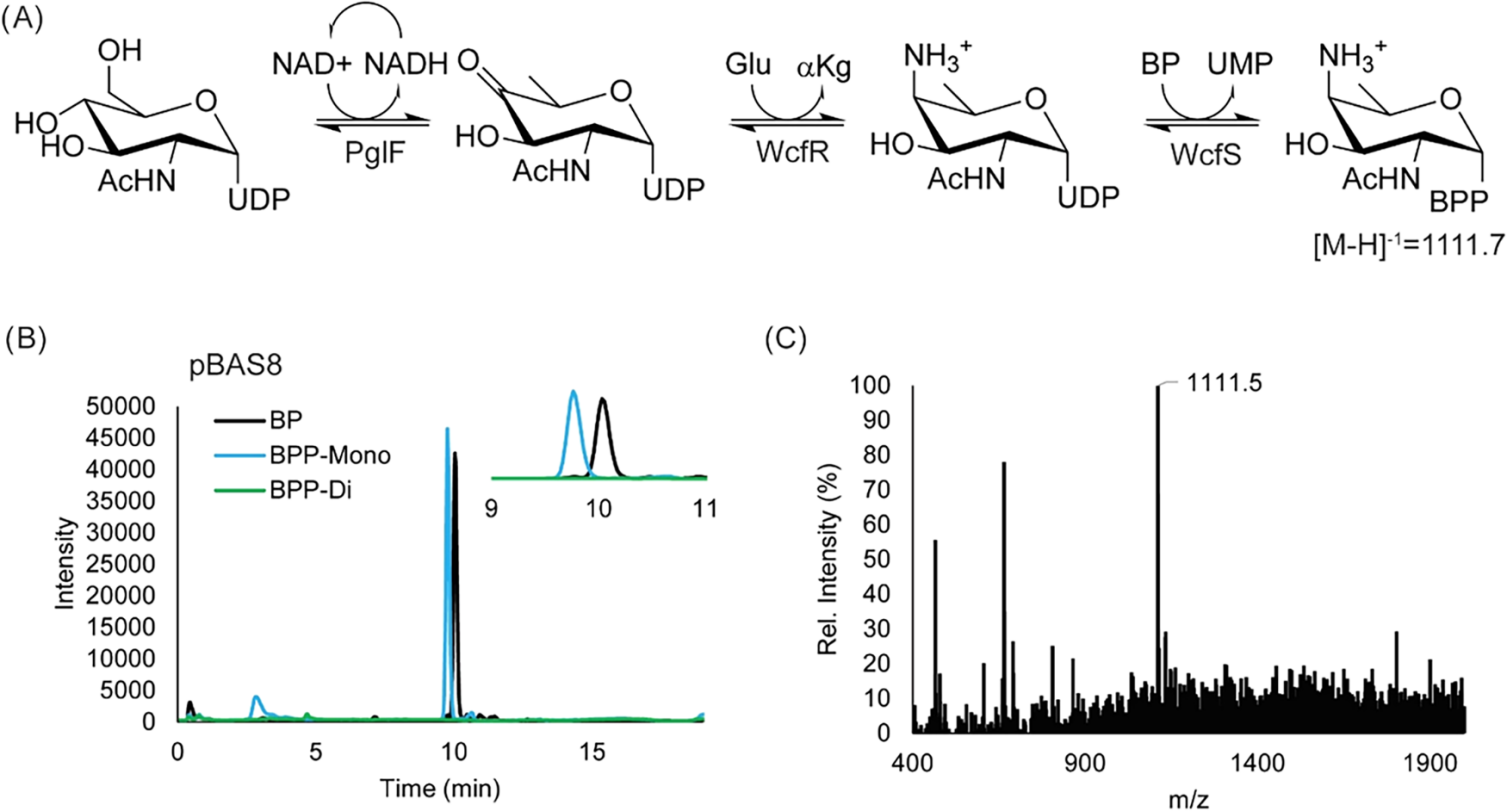
Formation of BPP-AATGal with pBAS8. (A) Reaction pathway for the generation of UDP-AATGal and transfer to BP. UDP-*N*-acetyl-glucosamine is converted to UDP-4-keto-6-deoxy-*N*-acetyl-glucosamine via the *C. jejuni* dehydratase, PglF. Then, UDP-4-keto-6-deoxy-*N*-acetyl-glucosamine is aminated via the *B. fragilis* enzyme, WcfR. Finally, AATGal-P is transferred to the BP lipid anchor by WcfS. (B) LC-MS SIM chromatogram scanning for BP (black), BPP-AATGal (blue), and the next potential product, BPP-AATGal-Gal (green) of pBAS8 expressing cells. The inset shows resolution of the two peaks with the shift in retention corresponding to the addition of AATGal-P to BP. (C) Mass spectrum of the peak corresponding to BPP-AATGal which shows a signal for the [M-H]^−1^ ion 1111.5.

**Table 1:** Plasmids Used to Construct CPSA Repeating Unit^a^

			expected	observed	
plasmid	geneslncorporated	CPSA intermediate	[M-H]^−1^	[M-H]^−1^	[M-2H]^−2^
pQE80L	BP	845.67			
pBAS8	*pglF*_*Cj*_, *wcfRS*	BPP-AATGal	1111.73	1111.5	
pBAS9	*pglF*_*Cj*_, *wcfRSQ*	BPP-AATGal-Gal	1273.76	1273.6	
pBAS10	*pglF*_*Cj*_, *wcfRSQO*	BPP-AATGal-PyrGal	1343.79	1343.6	671.4
pBAS11	*pglF*_*Cj*_, *wcfRSQOP*	BPP-AATGal-PyrGal	1343.79	1343.6	671.4
pBAS12	*pglF*_*Cj*_, *wcfRSQOPMN*	BPP-AATGal-PyrGal	1343.79	1343.6	671.4
pBAS15	*pglF*_*Cj*_, *wbpP*_*Vv*_, *wcfRSQOP*	BPP-AATGal-PyrGal-GalNAc	1546.87	1546.7	773.3
pBAS16	*pglF*_*Cj*_, *wbpP*_*Vv*_, *wcfRSQOPMN*	BPP-AATGal-PyrGal-GalNAc-Galf	1707.92		854.3

a Each plasmid incorporates genes from the organisms *C. jejuni* *(Cj)*, *B. fragilis*, and *V. vulnificus* *(Vv)* to produce each BPP-linked CPSA intermediate, including the complete BPP-linked repeating unit (pBAS16).

#### Formation of a Pyruvylated BPP-Linked Disaccharide

After the addition of AATGal-P to BP, the next sugar to be incorporated is pyruvylated galactose, which provides a negative charge to the zwitterionic polysaccharide. The genes *wcfQ* and *wcfO* are known to encode the galactosyltransferase and pyruvylating enzymes, respectively (Figure 3). We next incorporated each of these genes into the pBAS8 plasmid (Table 1, **pBAS9/10**), then analyzed transformed DH5α for the formation of the BPP-linked disaccharide and pyruvylated disaccharide (Figure 3b–e). We again found that the isoprenoid-linked disaccharide and pyruvylated disaccharide were formed based on the retention time of the new products and detection of the expected ions for BPP-AATGal-Gal, [M-H]^−1^ 1273.6 (Table 1, Figure 3b,c) and BPP-AATGal-PyrGal [M-H]^−1^ 1343.6 and [M-2H]^−2^ 671.4 representing the −1 and −2 ions for the pyruvylated species (Figure 3d,e). These products were not observed with an empty vector (Supporting Figure S2), nor were they observed without the appropriate genes incorporated into the vector.

**Figure 3:**
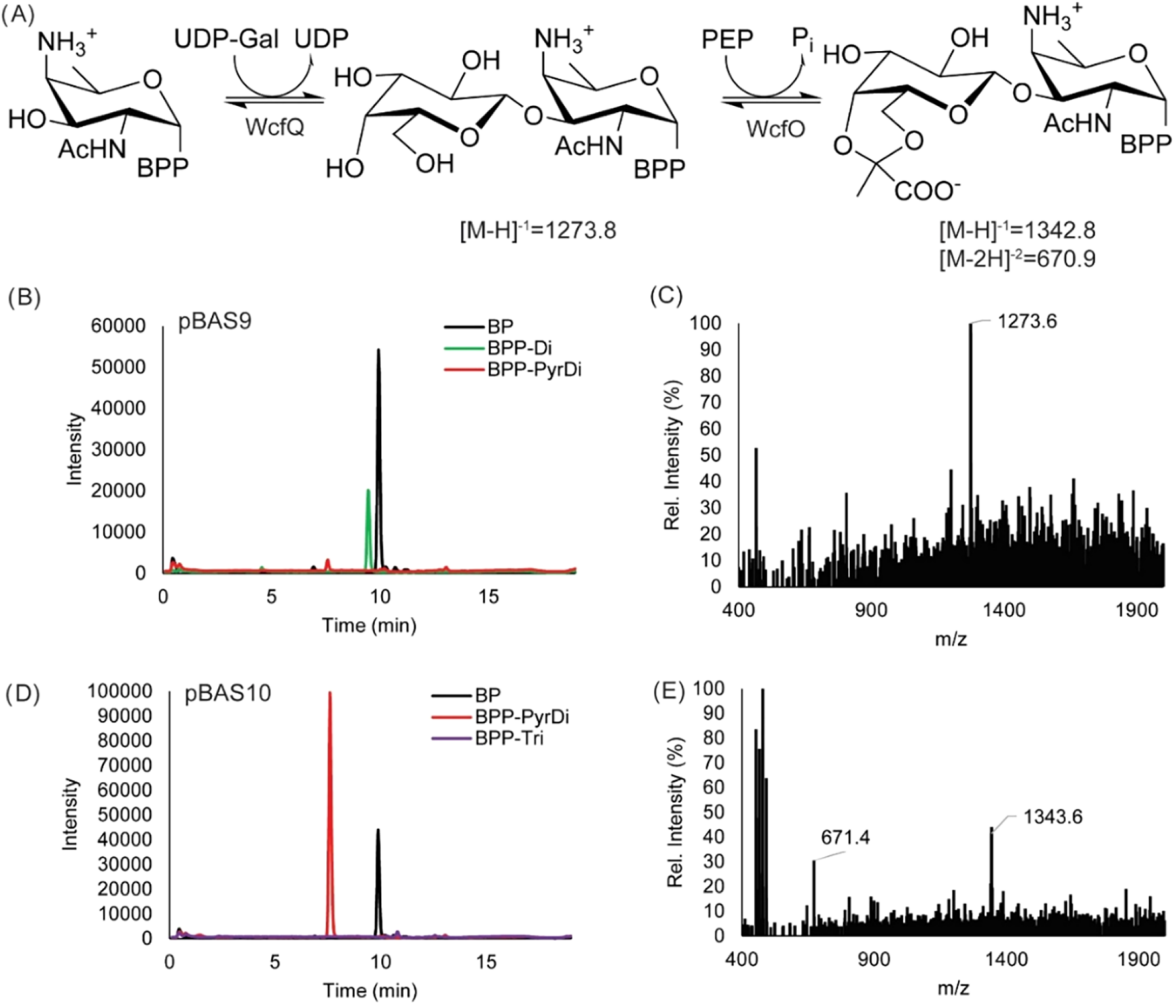
Formation of a BPP-linked disaccharide with pBAS9/10. (A) Reaction pathway with WcfQ transferring galactose from UDP and pyruvylation of galactose by WcfO using phosphoenolpyruvate (PEP). (B) LC-MS SIM chromatogram for BP (black), BPP-AATGal-Gal (green), and BPP-AATGal-PyrGal (red) of pBAS9 expressing cells. (C) Mass spectrum for the peak associated with BPP-AATGal-Gal (*m*/*z* 1273.6). (D) LC-MS SIM chromatogram for BP (black), BPP-AATGal-PyrGal (red), and BPP-AATGal-PyrGal-GalNAc (purple) displaying a new retention time with addition of pyruvate to galactose of pBAS10 expressing cells. (E) Mass spectrum of the BPP-AATGal-PyrGal peak showing signals for the −1 and −2 ions (1343.6 and 671.4, respectively).

#### Addition of a 4-Epimerase is Critical for CPSA Trisaccharide Formation

Trisaccharide formation requires the addition of GalNAc to BPP-AATGal-PyrGal (Figure 4). Therefore, we next constructed a plasmid that added the GalNAc transferase encoding gene *wcfP* (Table 1, pBAS11). We were surprised to find that no isoprenoid-linked trisaccharide formed, and we observed only the disaccharide product of WcfO (Figure 4b). Closer inspection of the DH5α genome indicated that this strain does not encode a clear GlcNAc-4-epimerase that could convert UDP-GlcNAc to UDP-GalNAc. Our previous work demonstrated that WcfP could utilize UDP-GlcNAc to a limited extent as a substrate; however, since no product containing GlcNAc was observed, it was clear that this limited activity was not relevant *E. coli*.^35^ We have recently shown that the gene *wbpP* from *Vibrio vulnificus* encodes a UDP-GlcNAc 4-epimerase and is robustly expressed in *E. coli*.^36^ We incorporated *wbpP* into our plasmid (pBAS15) and found that indeed the lack of UDP-GalNAc was the likely reason that we could not detect BPP-trisaccharide without it. The BPP-trisaccharide was readily detected in this strain with LC-MS SIM [M-H]^−1^ = 1546.7 and [M-2H]^−2^ = 773.3 (Figure 4c,d). No peaks with these *m*/*z* values were formed without *wbpP* present or with the empty vector (Supporting Figure S2).

**Figure 4:**
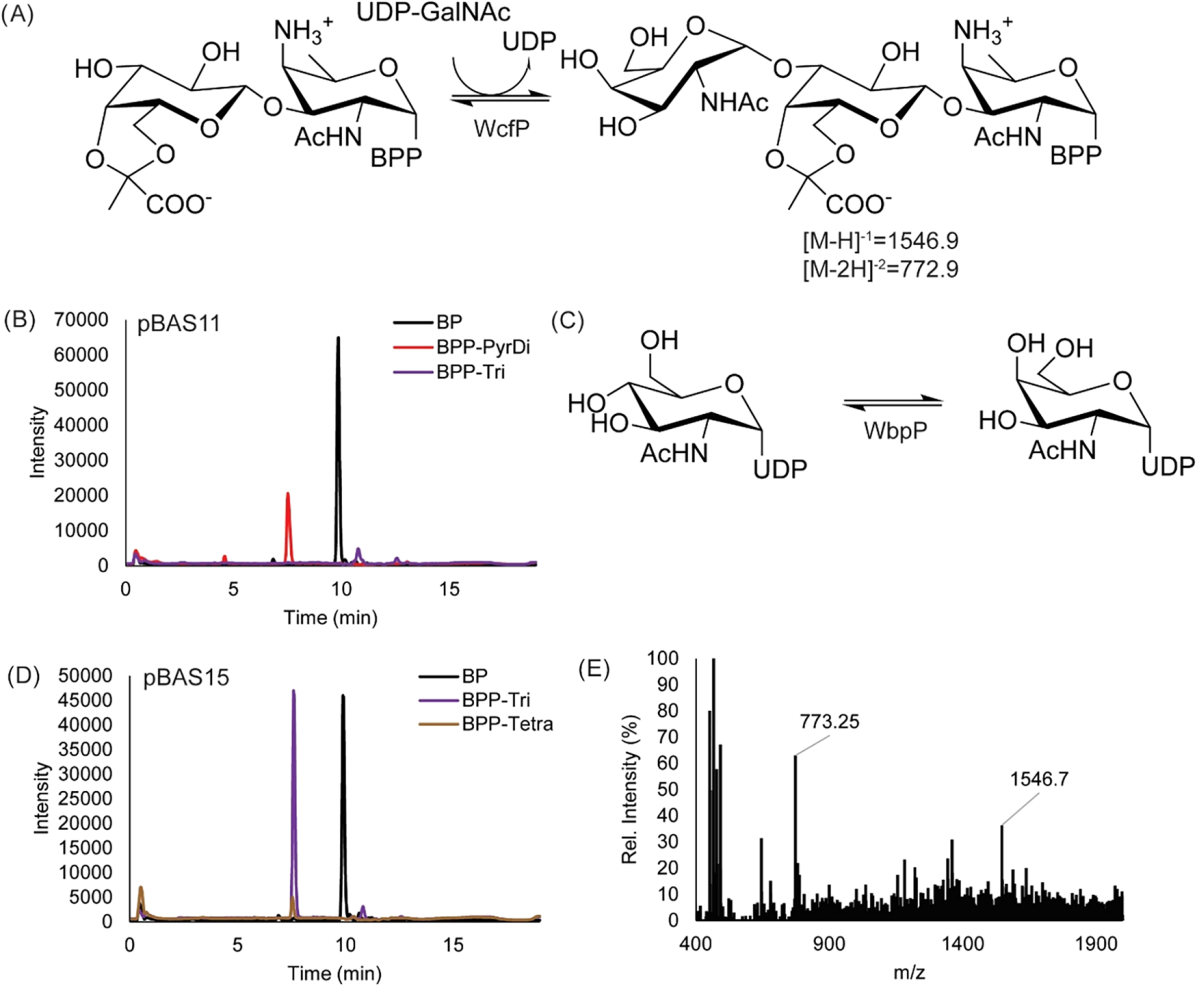
Formation of BPP-linked trisaccharide with pBAS15. (A) Reaction catalyzed by WcfP. (B) LC-MS SIM chromatogram scanning for BP (black), BPP-AATGal-PyrGal (red), and BPP-AATGal-PyrGal-GalNAc (purple) in cells expressing pBAS11. No trisaccharide-linked BPP was observed. (C) Reaction scheme showing epimerization of UDP-GlcNAc to UDP-GalNAc by the *V. vulnificus* WbpP. (D) LC-MS SIM chromatogram of BP (black), BPP-AATGal-PyrGal-GalNAc (purple), and BPP-AATGal-PyrGal-GalNAc-Gal*f* (brown) in pBAS15 expressing cells showing the presence of the expected product of WcfP with WbpP present. (E) Mass spectrum of the BPP-AATGal-PyrGal-GalNAc peak from panel (D) demonstrating clear signals for the [M-H]^−1^ and [M-2H]^−2^ ions.

#### Complete Tetrasaccharide Repeat Unit Assembly

The final step in CPSA repeat unit assembly is the addition of Gal*f* by WcfN. The Gal*f* sugar is initially formed through the isomerization of UDP-galactopyranose to the furanose form by WcfM (Figure 5).^21^ We incorporated both the *wcfM* and *wcfN* genes to produce vectors pBAS12 and pBAS16, where pBAS12 did not include the epimerase encoding gene *wbpP* (Table 1). Lysates of transformed DH5α were then analyzed for complete repeat unit formation. A SIM peak corresponding to the [M-2H]^−2^ 854.3 was detected for the repeating unit (Figure 5b) that was also clear in the total ion mass spectrum (Figure 5c) and once again, only disaccharide was detected without *wbpP* (data not shown). We did not observe substantial product ions for the −1 charged species and noted that the intensity of the −1

**Figure 5:**
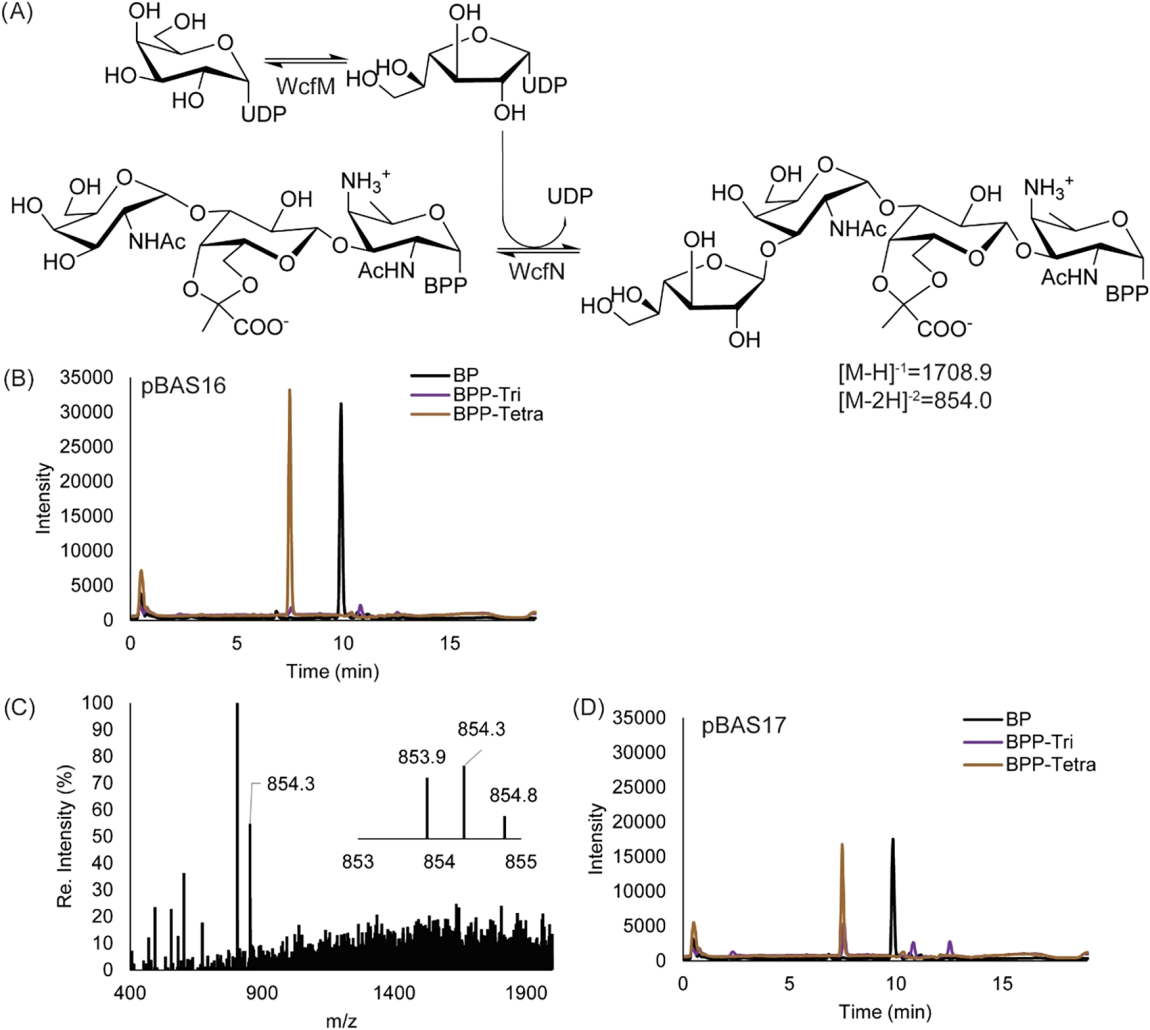
Formation of BPP-linked tetrasaccharide with pBAS16. (A) Reaction pathway for the formation of UDP-galactofuranose from UDP-galactopyranose by WcfM and transfer of Gal*f* by WcfN. (B) LC-MS SIM chromatogram for BP (black), BPP-AATGal-PyrGal-GalNAc (purple), BPP-AATGal-PyrGal-GalNAc-Gal*f* (brown) in extracted lysates of *E. coli* expressing pBAS16. (C) Mass spectrum of the BPP-tetrasaccharide from panel (B), showing a clear signal for the [M-2H]^−2^ BPP-AATGal-PyrGal-GalNAc-Gal*f.* Inset shows isotopic abundance associated with the −2 charged species. (D) LC-MS SIM chromatogram scanning for the same species in B in lysates of *E. coli* expressing pBAS17.

charged species for each product progressively decreased with larger glycans (Figures 2–5). We next incorporated genes encoding the predicted flippase (*wzx*) and polymerase (*wzy*) (pBAS17, Table 2) associated with the CPSA biosynthesis operon. In strains expressing pBAS17, we observed BPP-linked tetrasaccharide, indicating that if there was polymer formation, it did not utilize all of the available tetrasaccharide (Figure 5d).

**Table 2:** Plasmids Containing *B. fragilis* Polymerase or Flippase^a^

plasmid	*B. fragilis* flippase *wzx*	*B. fragilis* polymerase *wzy*
pBAS17	+	+
pBAS18	+	–
pBAS19	–	+

a Each plasmid includes *pglF*_*Cj*_,*wbpP*_*Vv*_, *wcfRSQOPMN* in addition to the indicated polymerase or flippase genes.

#### Coexpression of *wzx* and *wzy* from *B. fragilis* Produce CPSA Polymers

Once we confirmed production of the CPSA BPP-linked repeat unit by our recombinant *E. coli* strain, we were interested in whether polymer was formed in strains that contained pBAS17 which included the *B. fragilis* CPSA flippase (*wzx*) and polymerase (*wzy*) genes. In addition, we switched to an MG1655 strain of *E. coli* due to access to a variety of mutant strains that would allow us to interrogate polysaccharide formation. To evaluate whether CPSA polymer was formed in cells expressing pBAS17, we tested cell lysates by SDS-PAGE and blotting with anti-CPSA serum cleared of nonspecific antibodies through adsorption with *B. fragilis* that does not produce CPSA.^22,28^ We found polymers that were reactive with the anti-CPSA serum in MG1655 expressing pBAS17 at an apparent molecular weight of around 20 kDa relative to a protein standard (Figure 6). The growth medium also contained anti-CPSA serum reactive polymer. However, no anti-CPSA serum reactive polymer was detected with empty vector transformed cells.

**Figure 6:**
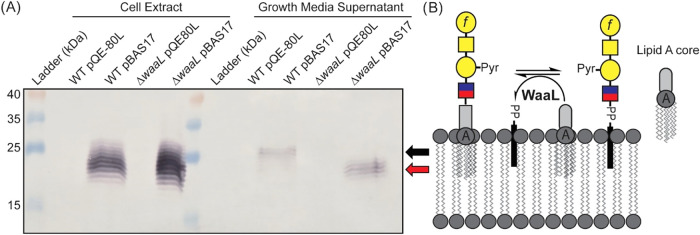
Formation of CPSA polymer detected by anti-CPSA serum. (A) Wild-type and Δ*waaL* MG1655 strains containing pQE-80L or pBAS17 were lysed and blotted with an anti-CPSA antibody serum. In the presence of pBAS17, polymer was detected both from cells and from the growth culture media supernatant from the cell culture. A small difference in molecular weight was observed between the Δ*waaL* deletion mutant (red arrow) and the wild-type strain (black arrow) in the growth media which was not clear in this cell extract. (B) expected transfer of the CPSA repeat unit to Lipid A by the WaaL ligase.

#### WaaL Ligates CPSA Fragments to Lipid A

In *E. coli*, it has been shown that oligo- and polysaccharides other than O-antigens (O–Ag) can be ligated to the lipid A core of LPS, including colanic acid, enterobacterial common antigen (ECA), and peptidoglycan fragments through the activity of the ligase, WaaL (Figure 6B).^25,26,37^ We next chose to test for the production of the CPSA polymer in a Δ*waaL* strain of MG1655. Interestingly, we observed that a slightly lower molecular weight polymer formed in the absence of *waaL* in the growth medium supernatant (Figure 6A). We suspect that the difference in molecular weight is due to CPSA being linked to lipid A by the WaaL ligase, while in the absence of WaaL, the polymer is not linked to the bacterial cell surface. The decrease in molecular weight was not as apparent in this analysis of the cell lysate, but was clear in the analyses below.

#### Native *E. coli* Gene Impact CPSA Polymer Formation

We next chose to inspect the minimum requirements for recombinant glycan expression in MG1655. To do this, we separated Pronase treated cell lysates of Δ*waaL* (ligase), Δ*wza* (transporter), Δ*waaL*Δ*wza*, or Δ*wzxB* (O-antigen flippase) MG1655 expressing either an empty vector or pBAS17and analyzed by Pro-Q Emerald 300 stain which detects sugar polymers. We found that in wild-type MG1655 expressing pBAS17, there was a high molecular weight polymer formed just above the 26 kDa protein standard that was not present with the empty vector (Figure 7). We also observed this same sized polymer with the deletion of the *wza* and *wzxB* genes. However, as observed in our anti-CPSA serum blotting (Figure 6), when the *waaL* gene was deleted, a lower molecular weight polymer was formed with an apparent molecular weight lower than the polymer formed in strains without this deletion. In addition to the bands detected at an apparent molecular weight relative to protein standards at 26 kDa and slightly smaller than 26 kDa, we also detected two unique bands with a molecular weight slightly higher than that of lipid A (approximately 15 kDa relative to protein standards) that were not present when the *waaL* gene was deleted or in the empty vector control strain (Figure 7). These bands were unaffected by the absence of *wza* or *wzxB*. We suspect that these bands represent one or two repeat units linked to lipid A through the activity of the ligase.

**Figure 7:**
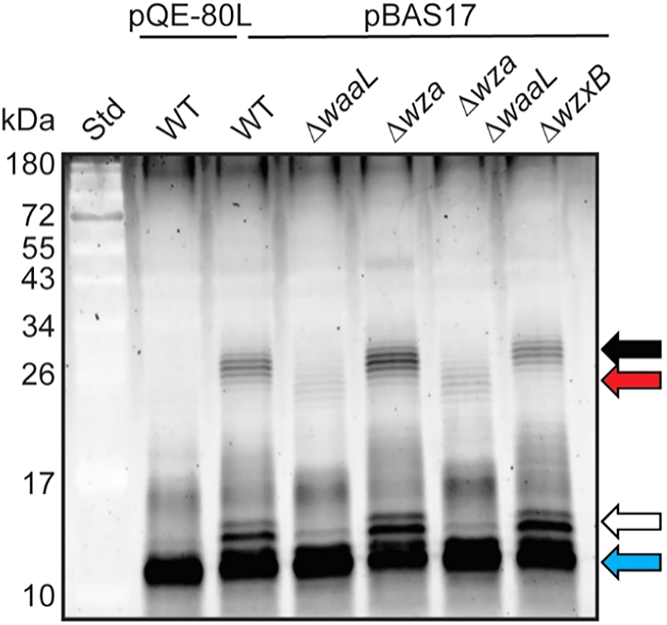
CPSA is linked to Lipid A through the activity of WaaL. Pro-Q Emerald 300 stain of LPS from Pronase treated cell lysates show production of a higher molecular weight polymer with pBAS17 in wild-type (black arrow) MG1655 compared to Δ*waaL* (red arrow). Deletion of *wza* had no impact on materials formed relative to wild type transformed with pBAS17. Deletion of *waaL* resulted in loss of both lower molecular weight, presumed lipid A conjugates (white arrow) that are slightly higher molecular weight relative to lipid A (blue arrow). Deletion of the O-antigen flippase, *wzxB*, had no impact on glycan formations relative to wild-type MG1655 transformed with pBAS17. Empty vector transformed cells produced no high molecular weight polymers or presumed lipid A-CPSA conjugates.

#### *Escherichia coli* WzxB Promotes Formation of CPSA Polymers

It was not clear from the previous experiments whether *B. fragilis* flippase and polymerase were both required for CPSA polymer formation. To determine whether both *B. fragilis* enzymes were necessary for CPSA polymerization, we constructed plasmids containing only *B. fragilis* flippase (*wzx*) (pBAS18) or polymerase (*wzy*) (pBAS19, Table 2). We then evaluated CPSA production in wild type (wt), Δ*waaL*, and Δ*wzxB* MG1655 expressing pBAS16 (Table 1), 18, or 19 using anti-CPSA serum or Pro-Q Emerald 300 stain. MG1655 transformed with pBAS16 or pBAS18, which did not contain the *B. fragilis* polymerase, led to the formation of no anti-CPSA serum reactive polymers regardless of the strain background (Figure 8). However, pBAS19, which contained the polymerase but not the flippase, resulted in a polymer similar to pBAS17 in the wild type and Δ*waaL* backgrounds. Interestingly, no polymer was observed in Δ*wzxB* MG1655 with pBAS19 suggesting that there was not another flippase in *E. coli* capable of transporting the repeat unit through the inner membrane. We next inspected the glycoprofile of these strains (Figure 8b). We found that with pBAS16 (−*wzx*_*Bf*_, −*wzy*_*Bf*_), we observed a small molecular weight lipid A conjugate at approximately 13 kDa relative to a set of glycoprotein standards in the wild-type strain that was not present in the empty vector control. This band was also absent in the Δ*waaL* and Δ*wzxB* backgrounds with pBAS16. This indicated that the repeat unit was likely transferred via WaaL, and without the *B. fragilis* flippase, required the *E. coli* O-antigen flippase for transport across the inner membrane. In strains transformed with pBAS18 (+*wzx*, −*wzy*), we again observed no higher molecular weight polymers but did observe presumed lipid-A modified glycan in the wild-type and Δ*wzxB* strains, suggesting that the *B. fragilis* flippase could replace the endogenous WzxB protein. Finally, with pBAS19 transformed strains (−*wzx*, +*wzy*), we observed polymer consistent with the anti-CPSA serum blot with both the wild-type and Δ*waaL* strain, but without the *E. coli* *wzxB* or *B. fragilis* flippase, we did not observe any unique product relative to empty vector controls. It is important to note the presence of a presumed lipid-A modified material in wild-type strains transformed with pBAS16–19 indicating repeat unit transfer occurs only when *waaL* is present. It does not appear that the anti-CPSA serum is useful for detecting these likely single repeat units. It is also important to note that the Δ*waaL* mutant consistently leads to a lower molecular weight polymer formation when the *B. fragilis* polymerase is present in the plasmid. The difference in the molecular weight could be related to the addition of the polymer to lipid-A.

**Figure 8:**
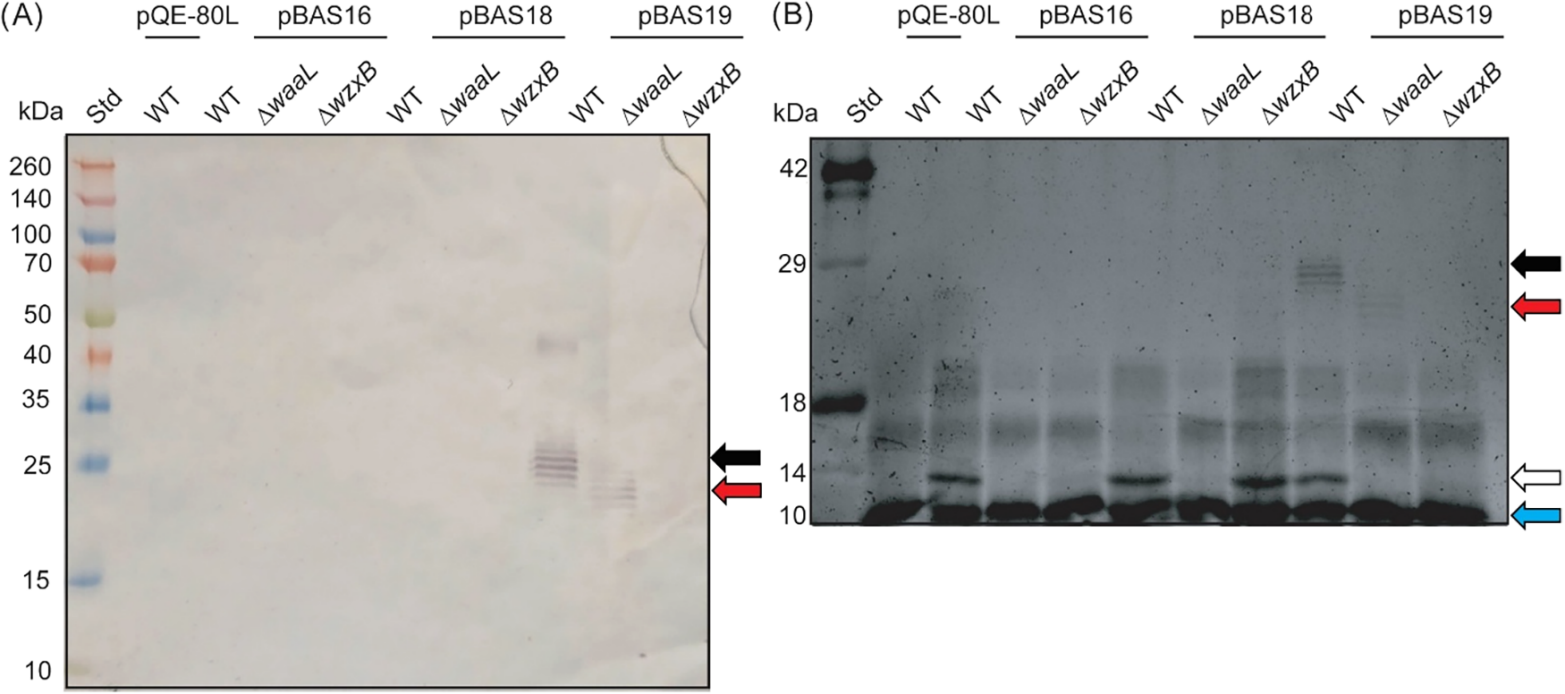
CPSA production in *E. coli* is dependent on a *B. fragilis* polymerase but not the flippase. (A) anti-CPSA serum blot of lysates from MG1655 strains (wt, Δ*waaL*, and Δ*wzxB*) transformed with pBAS16 (−*wzx*, −*wzy*), pBAS18 (+*wzx*, −*wzy*), pBAS19 (−*wzx*, +*wzy*) or the empty vector (pQE-80L). A sharp CPSA polymer ladder (black arrow) is detected when the *B. fragilis* polymerase is expressed from plasmid pBAS19 which contains only the polymerase. The downward shift in the Δ*waaL* lane confirms the ligase role in conjugating the polymer to the lipid A-core (red arrow). The complete loss of signal in the Δ*wzxB* lane with pBAS19 suggests that the native *E. coli* flippase is required if the Wzx flippase from *B. fragilis* is not included. (B) Pro-Q Emerald 300 stain of glycans from Pronase treated cell lysates identical to panel (A). Polymer consistent with the anti-CPSA blot was formed with a molecular weight of approximately 29 kDa (black arrow) relative to the CandyCane Glycoprotein ladder and a smaller molecular weight ladder was also detected (red arrow). Lipid A conjugates are also observed with pBAS16/18/19 in wild-type MG1655 (white arrow), but not Δ*waaL* or Δ*wzxB* except when the *B. fragilis* flippase is included with pBAS18. Lipid A is shown with a blue arrow. Note that we did not detect these lower molecular weight lipid A conjugates with the anti-CPSA serum suggesting that conjugate does not have an epitope for the anti-CPSA serum or detection required multiple epitope interactions to rise above detection limits.

## Discussion

A detailed understanding of the biochemical requirements for recombinant polysaccharide biosynthesis is essential for expanding access to structurally complex glycans, which are otherwise difficult to obtain. In this work, we reconstructed the biosynthetic pathway for capsular polysaccharide A (CPSA) from *B. fragilis* in *E. coli* and used this system to systematically evaluate individual steps of the Wzx/Wzy-dependent polysaccharide pathway.^38,39^ Such approaches provide a practical alternative to total chemical synthesis, direct isolation from *B. fragilis*, or chemoenzymatic production strategies, which remain limited by the lack of a purified CPSA polymerase capable of *in vitro* polymerization. More broadly, this work illustrates a general framework for dissecting bottlenecks in recombinant polysaccharide production systems.

Reconstitution of CPSA biosynthesis in *E. coli* revealed that production of the repeating unit requires supplementation of enzymatic activities not encoded within the native CPSA biosynthesis operon. Incorporation of a UDP-GlcNAc C4 epimerase and a 4,6-dehydratase, which are absent from the CPSA operon but required for precursor sugar biosynthesis, was necessary to generate detectable CPSA intermediates.^24,40^ These results highlight a recurring limitation of heterologous glycan expression systems: biosynthetic gene clusters often rely on host metabolic enzymes that may not be present or sufficiently expressed in the recombinant host. The stepwise reconstruction strategy used here provides a systematic means of identifying these requirements.

This system also enabled evaluation of key steps of CPSA assembly in the recombinant host. Surprisingly, CPSA polymerization was dependent on expression of the *B. fragilis* Wzy polymerase, whereas the native *E. coli* flippase WzxB could functionally substitute for the CPSA flippase Wzx_*Bf*_.^41^ The observation that CPSA polymers were produced in the absence of Wzx_*Bf*_ indicates that WzxB possesses sufficient substrate tolerance to translocate CPSA isoprenoid-linked oligosaccharides. This promiscuity is consistent with previous reports of relaxed specificity among Wzx flippases and suggests that endogenous flippases in *E. coli* may support the production of a broad range of heterologous glycans.^37,42^ At the same time, the ability of *wzx*_*Bf*_ to complement a Δ*wzxB* mutant demonstrates that the *B. fragilis* flippase is functionally compatible with *E. coli*.

Despite evidence for functional flippase and polymerase activity, accumulation of BPP-linked tetrasaccharide intermediates was observed in strains expressing both Wzx and Wzy. The persistence of these intermediates suggests that polymerization by Wzy_*Bf*_ may represent a limiting step in the recombinant system. One explanation is that efficient CPSA polymerization requires additional components of the native capsular transport machinery, such as Wza and Wzz, which are thought to assemble into a complex that coordinates polymerization, export, and chain length regulation.^43^ Alternatively, inefficient expression or folding of the multipass membrane protein Wzy_Bf_ in *E. coli* could also limit catalytic turnover and lead to accumulation of lipid-linked intermediates.

Consistent with this interpretation, the CPSA polymers produced in the recombinant system were substantially shorter than those isolated from native *B. fragilis*. Native CPSA typically appears as a 180 kDa polymer by SDS-PAGE, whereas the polymers produced here were markedly smaller.^28^ Previous studies have shown that CPSA fragments exhibit size-dependent biological activity: short oligomers (∼5 kDa, six repeating units) lack protective activity in mouse intra-abdominal abscess models, whereas fragments containing ∼22 repeating units (∼17 kDa) exhibit activity comparable to native CPSA.^44^ Structural studies further indicate that as few as three repeating units can form the helical conformation necessary for MHCII presentation.^40^ These observations suggest that even modest improvements in polymer length within recombinant systems could yield material suitable for functional and immunological studies.

The accumulation of lipid-linked CPSA intermediates may also impose physiological constraints on the host cell.^45–49^ Cells expressing CPSA constructs exhibited irregular morphologies and altered growth profiles following induction (data not shown). Similar phenotypes have been reported in other glycan biosynthesis systems when bactoprenyl phosphate (BP) becomes sequestered in stalled intermediates, thereby limiting its availability for peptidoglycan synthesis. Detection of BPP-linked CPSA tetrasaccharide intermediates in this study is consistent with such sequestration and highlights the importance of balancing glycan assembly with host lipid carrier availability when engineering recombinant pathways. Efficient recovery of soluble CPSA further required the disruption of native glycan attachment pathways. Deletion of *waaL*, which encodes the O-antigen ligase responsible for transferring glycans onto lipopolysaccharide, was necessary to obtain CPSA that was not covalently associated with lipid A. Similar strategies have been used in other recombinant polysaccharide systems to prevent diversion of heterologous glycans.^17^

## Conclusions

Collectively, these results underscore the challenges associated with transferring complex polysaccharide biosynthesis loci into heterologous hosts. Even when complete gene clusters are introduced, productive glycan synthesis may depend on host metabolic enzymes, membrane transport systems, and regulatory factors not encoded within the cluster itself. Recent efforts to engineer *E. coli* strains optimized for recombinant glycan production including modifications that enhance precursor availability, control polymer chain length, and minimize competition from endogenous pathways provide promising avenues to address these limitations.^18,45^ The modular strategy described here complements these approaches by enabling the systematic identification of bottlenecks within recombinant Wzx/Wzy-dependent pathways. Integration of these strategies should facilitate scalable production of structurally defined glycopolymers and expand access to biologically important polysaccharides, such as CPSA.

## Supplementary Material



## Notes

Primers used for preparing plasmids and *E. coli* mutants and strain genotypes, the sequence of the full pBAS17 plasmid obtained through whole plasmid sequencing (Eurofins-Operon), and LC-MS chromatograms for blank pQE-80L with each intermediate SIM (PDF)
