# Supplementary material for: Stepwise Assembly of the Bacteroides fragilis Capsular Polysaccharide A Repeating Unit in Escherichia coli
Source: Biochemistry. 2026 Mar 25;65(7):1013–24. doi: 10.1021/acs.biochem.6c00056 (PMC13063421; doi:10.1021/acs.biochem.6c00056)
Supplement: Supplementary file 1 [file bi6c00056_si_001.pdf]

Supporting Information

Stepwise assembly of the *Bacteroides fragilis* Capsular Polysaccharide A repeating unit in *Escherichia coli*

Beth A. Scarbrough[a], Claire E. Moneghan[a], Sara Salamat[a], Manoj K. Dooda[b], Alexis H. Murray[a], Jenna S. Costelloe[a], Matthew A. Jorgenson[c], Jerry M. Troutman[a]*

Department of Chemistry[a] University of North Carolina at Charlotte, 9201 University City Blvd., Charlotte, NC 28223, USA.

Department of Biological Sciences[b] University of North Carolina at Charlotte, 9201 University City Blvd., Charlotte, NC 28223, USA.

Department of Microbiology and Immunology,[c] University of Arkansas for Medical Sciences, 4301 W. Markham St., Little Rock, AR, USA 72205

* **Jerry M. Troutman** - *Department of Chemistry, University of North Carolina at Charlotte, 9201 University City Blvd., Charlotte, North Carolina 28223, United States*; https://orcid.org/0000-0002-8340-462X; Email: jtroutm3@charlotte.edu

Figure S1. Sequence of the pBAS17 plasmid

TTGAGATCCTTTTTTTCTGCGCGTAATCTGCTGCTTGCAAACAAAAAAACCACCGCTACCAGCGGTGGTT
TGTTTGCCGGATCAAGAGCTACCAACTCTTTTTCCGAAGGTAACTGGCTTCAGCAGAGCGCAGATACCAA
ATACTGTCCTTCTAGTGTAGCCGTAGTTAGGCCACCACTTCAAGAACTCTGTAGCACCGCCTACATACCT
CGCTCTGCTAATCCTGTTACCAGTGGCTGCTGCCAGTGGCGATAAGTCGTGTCTTACCGGGTTGGACTCA
AGACGATAGTTACCGGATAAGGCGCAGCGGTCGGGCTGAACGGGGGGTTCGTGCACACAGCCCAGCTTGG
AGCGAACGACCTACACCGAACTGAGATACCTACAGCGTGAGCTATGAGAAAGCGCCACGCTTCCCGAAGG
GAGAAAGGCGGACAGGTATCCGGTAAGCGGCAGGGTCGGAACAGGAGAGCGCACGAGGGAGCTTCCAGGG
GGAAACGCCTGGTATCTTTATAGTCCTGTCGGGTTTCGCCACCTCTGACTTGAGCGTCGATTTTTGTGAT
GCTCGTCAGGGGGGCGGAGCCTATGGAAAAACGCCAGCAACGCGGCCTTTTTACGGTTCCTGGCCTTTTG
CTGGCCTTTTGCTCACATGTTCTTTCCTGCGTTATCCCCTGATTCTGTGGATAACCGTATTACCGCCTTT
GAGTGAGCTGATACCGCTCGCCGCAGCCGAACGACCGAGCGCAGCGAGTCAGTGAGCGAGGAAGCGGAAG
AGCGCCTGATGCGGTATTTTCTCCTTACGCATCTGTGCGGTATTTCACACCGCATATGGTGCACTCTCAG
TACAATCTGCTCTGATGCCGCATAGTTAAGCCAGTATACACTCCGCTATCGCTACGTGACTGGGTCATGG
CTGCGCCCCGACACCCGCCAACACCCGCTGACGCGCCCTGACGGGCTTGTCTGCTCCCGGCATCCGCTTA
CAGACAAGCTGTGACCGTCTCCGGGAGCTGCATGTGTCAGAGGTTTTCACCGTCATCACCGAAACGCGCG
AGGCAGCTCTAGATCATGCGCACCCGTGGCCAGGACCCAACGCTGCCCGAAATTCCGACACCATCGAATG
GTGCAAAACCTTTCGCGGTATGGCATGATAGCGCCCGGAAGAGAGTCAATTCAGGGTGGTGAATGTGAAA
CCAGTAACGTTATACGATGTCGCAGAGTATGCCGGTGTCTCTTATCAGACCGTTTCCCGCGTGGTGAACC
AGGCCAGCCACGTTTCTGCGAAAACGCGGGAAAAAGTGGAAGCGGCGATGGCGGAGCTGAATTACATTCC
CAACCGCGTGGCACAACAACTGGCGGGCAAACAGTCGTTGCTGATTGGCGTTGCCACCTCCAGTCTGGCC
CTGCACGCGCCGTCGCAAATTGTCGCGGCGATTAAATCTCGCGCCGATCAACTGGGTGCCAGCGTGGTGG
TGTCGATGGTAGAACGAAGCGGCGTCGAAGCCTGTAAAGCGGCGGTGCACAATCTTCTCGCGCAACGCGT
CAGTGGGCTGATCATTAACTATCCGCTGGATGACCAGGATGCCATTGCTGTGGAAGCTGCCTGCACTAAT
GTTCCGGCGTTATTTCTTGATGTCTCTGACCAGACACCCATCAACAGTATTATTTTCTCCCATGAAGACG
GTACGCGACTGGGCGTGGAGCATCTGGTCGCATTGGGTCACCAGCAAATCGCGCTGTTAGCGGGCCCATT
AAGTTCTGTCTCGGCGCGTCTGCGTCTGGCTGGCTGGCATAAATATCTCACTCGCAATCAAATTCAGCCG
ATAGCGGAACGGGAAGGCGACTGGAGTGCCATGTCCGGTTTTCAACAAACCATGCAAATGCTGAATGAGG
GCATCGTTCCCACTGCGATGCTGGTTGCCAACGATCAGATGGCGCTGGGCGCAATGCGCGCCATTACCGA
GTCCGGGCTGCGCGTTGGTGCGGATATCTCGGTAGTGGGATACGACGATACCGAAGACAGCTCATGTTAT
ATCCCGCCGTTAACCACCATCAAACAGGATTTTCGCCTGCTGGGGCAAACCAGCGTGGACCGCTTGCTGC
AACTCTCTCAGGGCCAGGCGGTGAAGGGCAATCAGCTGTTGCCCGTCTCACTGGTGAAAAGAAAAACCAC
CCTGGCGCCCAATACGCAAACCGCCTCTCCCCGCGCGTTGGCCGATTCATTAATGCAGCTGGCACGACAG
GTTTCCCGACTGGAAAGCGGGCAGTGAGCGCAACGCAATTAATGTAAGTTAGCTCACTCATTAGGCACAA
TTCTCATGTTTGACAGCTTATCATCGACTGCACGTAATCTAGAGGGCGGATTTGTCCTACTCAGGAGAGC
GTTCACCGACAAACAACAGATAAAACGAAAGGCCCAGTCTTTCGACTGAGCCTTTCGTTTTATTTGATGC
CTCAAGCTAGAGAGTCATTACCCCAGGCGTTTAAGGGCACCAATAACTGCCTTAAAAAAATTACGCCCCG
CCCTGCCACTCATCGCAGTACTGTTGTAATTCATTAAGCATTCTGCCGACATGGAAGCCATCACAAACGG
CATGATGAACCTGAATCGCCAGCGGCATCAGCACCTTGTCGCCTTGCGTATAATATTTGCCCATGGTGAA
AACGGGGGCGAAGAAGTTGTCCATATTGGCCACGTTTAAATCAAAACTGGTGAAACTCACCCAGGGATTG
GCTGAGACGAAAAACATATTCTCAATAAACCCTTTAGGGAAATAGGCCAGGTTTTCACCGTAACACGCCA
CATCTTGCGAATATATGTGTAGAAACTGCCGGAAATCGTCGTGGTATTCACTCCAGAGCGATGAAAACGT
TTCAGTTTGCTCATGGAAAACGGTGTAACAAGGGTGAACACTATCCCATATCACCAGCTCACCGTCTTTC
ATTGCCATACGAAATTCCGGATGAGCATTCATCAGGCGGGCAAGAATGTGAATAAAGGCCGGATAAAACT
TGTGCTTATTTTTCTTTACGGTCTTTAAAAAGGCCGTAATATCCAGCTGAACGGTCTGGTTATAGGTACA
TTGAGCAACTGACTGAAATGCCTCAAAATGTTCTTTACGATGCCATTGGGATATATCAACGGTGGTATAT
CCAGTGATTTTTTTCTCCATTTTAGCTTCCTTAGCTCCTGAAAATCTCGCCAAGCTAGCTTGGATTCTCA

CCAATAAAAAACGCCCGGCGGCAACCGAGCGTTCTGAACAAATCCAGATGGAGTTCTGAGGTCATTACTG
GATCTATCAACAGGAGTCCAAGCTCAGCTAATTAGAGCTTTAAAATTTGATTTTGGCATTAAACAAACAA
GTTAAGAAACAAAATATAGACATGAAAATAATAGAATTAATGGTCCCCCCATCTAGTAATACCTGGGAAA
AAGTTCCAATATAAAGTAACAAATTTAAGGTAGATTGTTTTCGTAAAAAATAATCAAACAATAACTCTGC
AACGAAAAAAACAAATATCATATTAATTATAGCCCCATATATTCCCCAATTAGCGTACCCTTCTGCCACC
AAACCAGGTTGAGCATAATCTGCACCAATCATATTTGCTGAAATTAATGGAAGGCTTACATAATCAAAAC
CTAATATAGGTGCAAAGCTCTTTACATATGTAATTCCCCATAAATATCCTTTCTGGTCAATATAATCTAT
TATATTCCCCCCCCAAATGTGACCAAATATCAAAAATACGATATGTTTGCCTAGATAACCAATTTTGCAA
TAATTGCGGCTCACTTAATACTTCAAACATATCATTGAAAGTATAGTTTCCATCAAAATTTGCATTTAAC
TTCATCATCTCTGAAATGATACCATACAAAATCGCTAAAGAACAAAACAATAGAAAAAAGCCAATAACTT
TATAACTTATTTTTTCTTTTCTTGAAAATAGCAATCCATTATTCAAAAACCAAATTAAAATAAAAGGGAA
CACATATTGAACGAATATAAACCGAGAAAATAATTGCGAAATAAGGATCAATACCGATGCAATAAAACCA
ATAATAAATACTACTATTTTCCTTGTAAAAGGATATTTCCAAATAGAAATGATAAAATAACTTGTAAAAG
GAACTAGAATTGTAGCATAGGACCTATTCCCTAATATCCATATAGGCAACAAGATTATAAAAAATGCCAT
CATTATTTTTCGAGCTTCTTGTCTTGTAGGCATGTATACCCATACTTTCTGCTCAATAGAAATTTTATCA
ATACATAAAAGATAAATATAAATAACAGTTGAAACAAAAATAAAAATAAGAAAGTATAAAGTGTAATGAT
CATTTGGTACACAAATATTAGGAAACCTCTGTATCAAATTCTGTATGTGAGAATTGTAAATAATTGCATA
TGGACCTACATTTTGAGCTAAAAAAGCCAAAAAGAAATAGACCAAATAACCTAGTGTTACTATTTTTACT
TTTCTTTGATATACATTTGTTACTAATAAAAAGACAAAAAACGAAATTATAAATAACAATACTCGAGAAA
GATTACTCCAATCAGAACTCGCTAAATTATAAACAAATACTATAAAAGATATAGCCTTAATAATAAAGAA
AGAAGTACTAGTCATATGTATATCCTCCTTCATTGTTTAAATATTGAAAGTAATAATTTCTTTAGTTGTA
ATGCGAGGGTATCTTTTATCATTAATAAATATGCGTAATAAACAATAACAGATAACAGTATACCTATCAA
TAAATAATTTACTTCATGTAAAGGGAAGACAAACAAAAAAGCTAATAGAATAGATATCACTATAGAACCT
ATTAGATAGTGTAAATTCTGCTTGGAGAGAATATTTATAGGTAAATATTTTCTTCCCAGCAAAATCATTA
TAACCGTTACCATAAATTCTGCGATAACAGTCCCAAGCGCCGCTCCATACTGCCCATATTGGGGTATTAG
TAAATAGTTTATAAGGAGATTTATACATGCTCCTACCATAGTAGATATAATTACGTACTTTTCTCTCCCT
TGCGGATACAATATTTGCATTCCAATAATTCCAGATAATCCAATAAATAAAACAATAGGTGCAACTAATT
TTAATGTCAAAATAGAAGGTTCAAAACCATTTCCACAAAAAATATGTATAATAGGTGCTGCCATAAAAAT
AAGTCCCACACTCATAGGAAGACTTAAAGCAATTGTAAAACTAGCAGCTTTATTTGCCAATAATTGGAAT
TCTTCTTTTTGACCATTAGTTATCATATTACTAAAACGAGGTAATAAAACAGCTCCTAATGAAGAAACTA
TACCCAAAATAGCTTTTGTAAGTCGAGTAGCAGCAGCATAATAACCAACTGATTCTTCATTTTTAAGAAA
TCCAAGCATCACAGAATCAAGATTCACATAAATACTTATAACCAAATTTAATATAAAAATTTTAAGTGCA
GGTATTAAATGCCTCAACAGATTCAAACGCTTAAATTCGCCTTTACTAAGTTTTATATATTTACGTAATC
GAAAAAAATTAAATATATTATTACCAACAGTACCAATAACTAATATTCCAGCATAATAAAACAGATCTTG
CTTGGTCTTAACAAAGATAAATAAGGCCACTAAAGATAGTATCCTTACAAATAAGGAACGTAAAGTTATG
TATTTAAAATCTTCAATTGCCTGATAAAACCATGCAACTCCTATCGTGTTAAAAAATAAAGTTGTACTTA
ACAAAAAGAAAAGAGAAGCATCTATTTCTATTTTGGCAACGGTCTTAGCAAGTATAAAAACAACTATATA
TCCGACTAAAGTCAAAATAGCATGAAGCAATAGTATTTCGATTGTTGTTCTACTTCTTAATTCCTTATTA
TCCCTTATTCTTGCAATTTCTCTAACGGCATACAAAGGGATTCCCAAAGCAGTACAAAGAGAGACATAGT
CAATTATGGATTGGAAAAATTGTACTTGCCCTATACCATCTGCCATTAATATACGTGAAGCATAGGGAAA
AGTTATTAGTGGAAATAATAGCCCAGTAATTGTAGTACTTAGATTCAGTAGGAAGTTATTCTTTATTGAT
TGTCCCATATGTATATCCTCCTTTAATAGGTAGCTCCATTTTTTTTACGAAAGCAATCATAAAAGCCACG
AAAAACAGATATTAAGTGTTTCATCTTATTATCATATAAAAAAATAGATAAATAAATCTGATGAACAAAT
TGATAAATATATTTAATATATAAAAACCTATTTTTCTTATCCAATAATAAATTAAGTGACTCATATACTA
AATGAAAATTCCGACATGCATAATAAGTATACATTGGGCTAACGCTACGATCCGATGATCTTCTAGCACG
AACCATGATAGCATCAGATACTAAAATTACGTTGGTATAAAAACTAGCTGCAAACCCATATATTGTATCA
TCTTCCGCTATAAAAAAACGCTTATCAGGAAATCCTATTTTATTAACAATGCTTTTCGAAACAAACATCC
CTTCAAAACAGCCTACATTAATAGCACAAAACTTTTTACCATTATTAAATGATTTTTCTTGCCAATAACC

AAATTTACTATAAGTAATTGGATCCAACCATTGTTCAAAATTTACTTCTACATCATCCGAATAATACCTA
CGTGGTTGGATACATTGAGAAATACTCGTCCACCCTAAGAGTTTTTCCAAACAATCTATATTAGGAAAAA
CATCATCATCCATCATCCAAATCCAATCTGCCTCATTCTCATATGCATATTTTACCCCCGTTTCGAATCC
ACCAGCCCCACCAGAATTAGATTGATTTATTAATGTTATATTTTTCTCCTGTTCTAACCATTCTTTTGTT
CCATCAGTAGAGCCATTATTTACTATAACTATACTATCTAATTGACGTGTCTGTCTTTTAAGTAAATCTA
TGACCTTTTTTAGTAATGCTAAACGATTATAGGTCACAACTACAGCAAATATCTTCATAAGTCACTATTT
ATAACTTTTTCCACAATCACATTCATATCATAATACTTATACTCAGCTAATCTCCCTCCAAACAAAACAT
TCTTCTCTCTATCGACCAATTTCTTATACTCCAAATACAAAGAATTATTCCTCTCATCATTAACAGGATA
AAAGGGTTCACTACCATCTTTCCATTCCGAAGAATATTCTTTGGAAATTACGGTTTTGGGCTGTGTTCCA
AATTCAAAATGTTTATGCTCTATAATACGAGTATAAGGCACTTCCCTTTCAGTATAATTCACAACTGCAT
TCCCTTGATAATTCTCACAATCAAGCACTTGTGTTTCAAATTCAACAGTACGATATTCCAATCTCCCAAA
TTGATAATTATAATATTCATCAATTTTGCCAGTAAATAGAATTTTATCCGCAAGACCTTTAAAATATTCT
TTATTTTGAAAAAAGTCTGTATTTGTTCTAACTTCAATACCATCTAATAAACCATTTATCAATTTATTAT
AGCCACCAACAGGAATCCCCTGATATTTATCATTAAAGTAATTATTATCAAAAGTAAATCTGACAGGTAA
ACGCTTGATTATAAAAGCAGGAAGTTCAGTTGCTTTCCGCCCCCACTGTTTCTCAGTATATCCTTTTATC
AATATTTCATAAATATCCTTTCCTACTAAAGAGATAGCCTGTTCTTCTAGATTCTTCGGCGTAAGAATCC
CCGCTTCTCTCCTTTGCTCTTCTATCTTCATTTTTGCTTCCTCTGGAGTTTTTGTCCCCCATAAAGCATA
AAAAGTATTCATATTAAAAGGTAAATTATATAATTTGCCTTTATAATTGGCAATAGGGGAATTAGTATAG
CGGTTAAATTCCACTATGGCATTTACAAAGTCCCAAACCTCTTTATTAGAAGTATGAAAAATATGTGCAC
CATATTTGTGTACATTTATACCCTCAATATTTTCGCAATAAATATTTCCTCCATTGTGTGGACGTTTGTC
AATAAGCAAACATTTTTTCCCATTCCGTTGCGCTTTATATGCAAAAACAGAACCATAAAGTCCGGCTCCG
ACAATTAGATAGTCATATTTTTTTTTCATATGTATATCCTCCTTTATTTTTTATCATTTATAAAGCTTAT
ATACCATGGCATTGCTTCGTTGATTCCATCTAGAATACGGTGCGACGGCGCATAGCCTAAATACTGCTTA
GCTTTATTAACATCAGCCTGAGAATGCCTTACATCACCAGTACGAAACTCTCTATAAATAGGTTCTTGAG
AAACAACAACAGTATTATTCGATAATGCCAATTTAATGGCAGAGAATAGATCGTTTAAGGTTGTTCTGTC
TCCTACTGCAACATTGTAAATCTTGTCTTTTGCTTCTTCTGGTGCGGTGGCGGCAAGAATATTCATTTGC
ACTGTATTAGCTATATAACAGAAATCGCGACTAGTCTCGCCGTCACCATTAATATAAATAACATCACCTT
GAATCATTGCTGCTGTCCATTTTGGAATAACAGCCGCATAGGCACCATTTGGATCTTGGCGTTTTCCAAA
CACATTAAAATAACGCAGTCCAATACTTTTAAAACCATAAGTCCGCGCAAATACATTCGCATAAAGTTCA
TTTACGTATTTGGTTACGGCGTAGGGGGATAACGGGTTGCCAATATTTTCTTCTACCTTCGGCAATGCAG
GGTGATCACCATAAGTCGAACTTGAGGCGGCATAAGTAAAGCTTTTCACTTGCTGATCTTTGGCTGCTAC
TAACATGTTTAAAAAACCAGTGATGTTAGCGGAATTTGTAGTAATAGGATCATTAATTGAACGGGGCACA
GACCCTAGTGCCGCTTGGTGTAATACATAGTCTACACCTTGAACCGCTGCTACACAGGTCTCAGGATTAC
GTATATCGCCTTCAATAAAGGTAAATCGTTGCCATTGGGCTTCTGTTACTAAACTTTTAACTTCATCTAA
GTTATGTTGATGACCCGTGGCAAAGTTATCTAACCCCACCACGGTTTGATCAAGCTTTAGTAGTTTTTCT
AGTAAATTGGAGCCAATAAAACCAGCAACACCGGTTATCAACCATGTTTTTGGTGATTCAACTAAATCTT
GTTGGATTTTTTCGTATTTGGTCATATGTATATCCTCCTTCATTTATGTAAAAGATTTAAATATATTCTT
TCGAGATATTCACTCTCTAAATGTATATCATATCCTGATTCACATATTTCTTTAGACGTATCTTTCCTGA
TAAAAGTTTTATCTTCAAACAACTTCCAAACAACTTCAGCCCAAGTTGAAGGATCTCGATTGAGATTAAG
ATAACTTATATTATTCGTTATCTTTGTTTCCAAAGTAATACGATCCGAAACGACACATCTTAAACCAGAA
GCTTGCGCTTCAATCAGAGTCACTGGTAGACCTTCAAAAAAAGAAGGAAAAATTAAAAAATCCATAGCTT
GTAATAATTCTGATATATCAGTTCTCCGACCTAAAAAGCACACATACTTCTGCAATCTTTTTAATTTTAC
TTTTTCCTCTATTTCACTTCTAAGTTGGCCATCCCCTACTAACAGCAACTTAACATTGTCATTAATATTC
AATAATTCTTCCAATATATCTATTAAAAAACCATGATTCTTTTCTTTCGTAAAGTTACCGATATGCCCCA
ACAGTAAACAGTCATTCTCTATTTTCAACTCAACTCTAACTCTCTCTCTAACAGTTGCATTATAAATAAA
TTTTTTAGAAAAAATTGCATTATGAATAATATAAACTTTTTCAAGTCTCTTTTTTCCAAAAAGGAATTCA
GCAGCTTTGTATCCACATGCAAAATAAGTGTTTGCTAAATATATTGTAGGAAATTGCAAAAAATAATTTC
TAATGGACCTTATTTTTTTACTAGAATATTTTGTACTGTGACTATGTAAAATCCTTATCCGTATTCCATA

TATCTTTGCAAATAAAAGGTCTAATACTCCTGTATTAGCACTATGTACATGAATTATATCATATTTTTTT
GCATTTCTCTTATAAAAACAGAAAATCACCTTTAAATATAAAAATAAATATTTCAAATGATATGAAGGGA
ATACAACAATATCGCTACCTAATTTTCTCACATAATTATCAAAATCTCCAATATTACTATCGGCAGAAAA
CACATATGTAAAATGGAATTTATTTAAATCAATAGAAGTGTTTATATTTTTCAATAAAGCACCAATTCCT
CCAACAGGTCTAGTCCCTACAATATGCAGTATTTCAATGGGCTTTCCATCCCTCATATGTATATCCTCCT
TCATGACATCATAAATTTATTACATATATTAATTAATCGATCCTCTATATTCTACAAAATCAATTTTATT
CTTAAAAGAAGAATCTCGTTTAAATAGTCCATCAAAAAAATTGGAAGTAACATTTTCCCCCTTCACAGTT
ATCACATTAAATAGAGATTCAAGTCCCCCTAATCGACAAGTGCTCTTCTTGCTTTCTGATAGATTTCTAA
TATAAACCACAGGGGTCTCAAAGCCTAAGCAAGGAAGAGCACAGTGGATTCTAGAAGTTATCACATATTG
AGCTGAGGCCATTTTAGTTAATAATTCATCAGCCCGAGCAAATCTTTCTGATTCAGAAGAATATTCATTA
GACATATTAAATTGTGTTATAAATATTGCATTGTATAATACGTCAGGAACTACTAGCTTTCTCAAAAGCA
AATAAGACTTGGTTATTAACAATAAACGTCCTATTCCTACTTTGGATATATTAATAGTAAATCTATTATT
CTTTTTATAATTTCTAAGAATCTTTAGCACAGGCTTCATATAAAAAACAGTTTGTACAACAGCTTTCATT
ATTTCAAAAAAATTATTCCCATTAGGCATGTATGAAAGTGGATCAACAATGTATATCCCCTCACGTTTTC
CATTATATTTATAAGTTTTACCCAAAGTAGTAGTAAGACATCCCGAAAAATAAGCATTAACACCTTTTCC
TTTTAAAATATTCATTGTATGATAATCGCGGCACCCTATGGGTTCATACTTTTTTAAATACTTAATACTA
TCATGTGACAAAAAGCGACTTTCTGCAGAAGAATTGATATGTAATGCAACAAATAATGGATAAACCTGAG
ATGAAGGAGGCCAATTATATGGTTTATAAGTCATCCAAGCATTCATTATGACTTTTGCAGGTTCCCCTTT
ATACAACTTTAATTCATCACGATTGAAAAATATATAATTATCATCATCAAAAAATTGTTTAGCGGCGATA
CTCTGAATATAATCACCAATATTTATGGTTTTAATATCTCCATATGTTAATAATATCTTCCTCATATGTA
TATCCTCCTTCAACGTACTGTTTTATAGATTATGTCAAGAATAGATTTAGGCATTAATTTGGCCATAGCA
TATAGCAATGCATAACAATCTTCCTTCCATCCGAACCCTAACATTCTATTAACACGATGACGTAAAGTAT
AAATAGATTTTGCATGTTTCCACCCCCGCCTTCTTTCAAAGAAGTTTGAATCCAATCTAAACTTAAACAG
ATATTCGGGTACATTGGCAAATTTACATCCACTTTTAAAACCTTTAGCCCACATCATAGTATCTTCTCCA
AAGTAGGTGTCTTCGGGATATAAACCAGCTTTCTCAAAATATGAACGTCGAAACATCACAGTAGGATGAA
TCATACAATCCCGCTTTTTAAACAGTTCCAAACATTCTTCATGAGTTATCGGCATTTTTTTTCGAAAATA
TTCTTTTCCGTCATCATCAATTTCTATTGCCCAAGTACCAAGGCAATCAATATCCGGATGTGAATTCATA
AATGCGATCTGCTTCACAAAACGTTCCGGCATGGAAATATCATCAGCATCCATACGAGCAATATATTCGT
ATTCTTTTGTAAGAACGATATTCAATAATTCATTTAGTGCATGAGCCAATCCTCTATTTTGTTCTTGCTT
AAATATATAAAGTGAGTTTTGATCAAATGAGTCTAAGAAATGCACAATATCAATCGAAACAGGACCATCA
CAATATAAATAAATATCACAAGTAACATTTTCTTGATTCAACAAACTTTGTAAGGATTGCTTTATTTTAG
AAAGCGAATCATTTTTGTAGATAGAAGAAATGACAGCCAATTTCATATGTATATCCTCCTGAGCTCCTAA
CGAATGATGCTCCAAAATGTCAGAAATATAATTTTAAAATATTCCTTCACCGAACGATTACAGATATATT
TCATATTATACCGTATCTTATCGGGCATAATCTGTTCTATATATGCTCTGTCAGGATCCGAAGCCTCTCC
AAGAATCATATTTTCATCTACATACTCAATAGAAGCATAATCCGTAATCCCCGGACGTACACTCAATACT
TTCTTTTGTTCATCGGTATATAAATCCACATACTTACGAACCTCAGGACGGGGCCCTACCAGACTCATAT
CTCCTTTCAGTACGTTAAACAACTGAGGCAATTCATCCAATTTATACTTCCGGATCAGGTATCCGGTACG
AGTTATTCTCGGATCTCTCCCTCCTACCGTTATCAACCCCTTTTTATCAGCCCCTACACGCATCGAACGA
AATTTATAAACATAAAAATCCCCTCCGTACCTTCCCACACGGAGCTGTTTATAGAAACCACCTCCTTTAC
TTTCGAGACAAATGGCTATATAAAGAAGAAGAAAAACGGGAGAAAGCAGAAGAATTCCCAAAAGGAAAA
AACGATATCAAAAAAACGGATCAAATGTATATCCTCCTCTAGTGGTGGTGGTGGTGGTGTCGGTTTTCTG
CAATTACGGTCGCATAGGCCGCCTTTACGGTTTCAATAATGAAATTCAATTTTTCACTATCCAATTGGGG
ATATATCGGCAATGATATCTCGCTCTTAAAGTTCTGATAAGCCTGTGGATAATCCTTTATATCATATCCC
ATCGATTTGAAGAACGACAACATCGGCATCGGTATAAAGTGCACATTGACAGCCACTTCACTTTTAGCGA
TTTCATCAATCATCCGGTCGCGTTGTTCTTCGGTAAAGTCCTTAATACGCAAAGCATAAATGTGATAAGA
GCTTTCCTTTTCCCCATCCACAGAAGGCGGAACGATGGCCCAGTCACAAGCCGAAAAAGCATCGCTATAA
GCATTAAACACCCGCTTTCGTTCTTTCAGCAATTCCGGATATTCTCGTATCTGTGCCAGTCCTATGGCGG
CATTTACATCGGCCATATTGATTTTCATCCCGAAACCGACAATATCATAACGCCATCCTCCGGCTTTAGA

TTTCGAAAAAGCATCCTTGGTCTGGCAGTTCAAGCTTGTCATCCGTAGCTCTTTATACAACTCCGTATTA
TCGAACGGCTTAGGCAGATTCAGGCAAATGGCCCCACCTTCGGCAGTAGTCACATTTTTCACGGCATGAA
GTGAAAAGATAGCCACATCAGTTTCACAACCGGTACGCTGACGGCTACTGTAACGTGCTCCCAAAGAGTG
GGCAGCATCATTCATCACCAAGATGCGTCCCAATTTTTCTTGCACAGGAGATTCCGAACGGAACAGTTTT
ACCATTTCTGGTTCCTGCACCAGTGCCATGATTCTTTCATAATCGCAGGGAAAACCAGCAATATCTACGG
GTATAATCGCCTTTGTTTTAGGAGTAATAGCTTTACGAACAGCCTCCACCGAAATATTAAAATCAGTTCC
GGAATCCACCATTACCGGTTTGGCTCCGGCATGGAGCACAGCCAATGCCGTTGCACTGTAAGTATAGGCA
GGAACAATCACTTCATCGCCCTCCTTCACTCCCAGCCAACGCAACATCATAATAGCTCCTGATGTCCAGG
AATTGACACAAAGCACTTCTTTTGCTCCTGAGAAAGATTTAATTTCTTCCTCCAAAGCCTTCACTTTCGG
ACCGGATGTGATCCAACCGGAACGTAACGAATCAACGACTTCGTTGATGACCGCCTCGTCAATATAAGGT
GGTGAAAAAGGTATTTTCATATGTATATCCTCCTTTATACACCTTCTTTATTGTGTTTAAATTCAGGTAC
AATCTTTAATAAAGCCTCTGAAATATCTTCACATATTTGTAAATTTTCTATCTCTTTATTAAGCCAATCA
AGGTCAACCTTCTCATTCTTTGCTACAAAAATACTCTCATATTGTGTTTTAGCATCATTTTCATCAATCA
AAAGCTCTTCATAAAGCTTCTCACCTTTTCTTAAGCCTGTGATTTTAATTTCTAAATCATTGCGATTAGA
AAGTAAAAGCATTTTTTTAGCTAAATCTATGATTTTCACAGGCTTACCCATATCCAAAACAAAAAGTTCT
CCCCCTTTTGCGATAGCTCCAGCTTGTAAAACAAGTTGCACTGCCTCAGCCACAAGCATAAAATAACGCA
CTATATCAGGGTGCGTTAAAGTTAAAGGCTCATTATTGGCAATTTGTGCTTTAAATTTCGGTATCACACT
ACCACTAGAACCTAAAACATTACCAAAACGCACACAAGCAACTTCAAAATTTTCATCACTCATACTTAAA
GTATAAAGCTCGCAAACTCTCTTAGTGCAACCCATAATATTTGTTGGTCGTACTGCTTTATCTGTACTTA
TCATCACAAATTTAGCTACTTTGTTTTCTTTAGCACTGTCGCATAAAATTTTAGTTCCTAAAATATTATT
GATTACTGCTGAATGTGGATTTTGTTCGCAAAGAGGCACATGTTTATAAGCGGCTGCATGTAAAATAAGC
TCGGGTTTATAAGTTTTTAATACCTCATCTAAACTTTGCTTATCTAAAATACTCAGTAAAATAGGAGTAA
TTTTTTCTTTATATAAATTTAAATCATCATTGATCTTATAAAGATTATACTCACTATGATCAACCATGAT
AAGATGCTTAGCACCAAATTTAATACATTGCTTACAAAGTTCACTGCCTATAGTTCCACCTGCTCCACTT
ACCAAAACTACCTTATCTTTTAAAAAAGCCGCCACAGCACTATCATCTAAATCTTTTGGTTTTCTAGCAA
GCAAGTCTTCTATACTGATATCTCTTGCTTCGTTTCTTGTAAAAGAAAATATTTTTACATCGCAAATACC
ATAAGCTACAAGTTCTTCAAAAAGTTTTTTAAGCTCTTCTTGTTCAAGTCTTAAAGCAATAATGGCAGTT
TTTACCCCTTGTTCTACATAAGATTTTATTTTTTCTTTTTCTTCTACAATAAATTTATCACAATAAGTCC
CTATAAGCTCTTTTCTCGCATCAACTACGCCTACAGGAAAAAGCCCTAAAGAACCTTCTTTTGCACCTTT
TAACAAATGCAAAGCCTTAGAAGTTGCCCCTACTACAATACAAGGAGTTTCTTCTTCTTTCATTCTAGAA
GGTTTAAAATCCACAAGCATTCTTTTGCTAATTCTTAAAGTACCTATAAACATATAAGAAAGAACAAAAT
CTATCACAATAGCACTTCTTGGAAAAGGATTAAAAAAATCACTAAAAAAATAAAAAATAAGAAAAAAACA
AAACTCAGCTAAAAGCAAAGCGATAAAAATCTTTCTTGCTTCATTGAGAGAAAAAAATCTCCAAGCTACT
TTATAAATTCTAAAAACAAACAAAAATGAAAGTTTTAAAACAAGCAAAATAATAGCAGAAACCATCATAC
CATGATAAAAAATACTCGGAATATCTCCACTAAATCTCAAAGAAAAAGCCAGATAAACGCTAAGTAAAAT
TAAAACAATATCTGAAGTTAAAAAAAATGCTAATCTTTTGCTTTTATAAAAAATCATTCCGTGATGGTGA
TGGTGATGCGATCCTCTCATAGTTAATTTCTCCTCTTTAATGAATTCTGTGTGAAATTGTTATCCGCTCA
CAATTGAATCTATTATAATTGTTATCCGCTCACAAAGCAAATAAATTTTTTATGATTTCTCGAGGTGAAG
ACGAAAGGGCCTCGTGATACGCCTATTTTTATAGGTTAATGTCATGATAATAATGGTTTCTTAGACGTCA
GGTGGCACTTTTCGGGGAAATGTGCGCGGAACCCCTATTTGTTTATTTTTCTAAATACATTCAAATATGT
ATCCGCTCATGAGACAATAACCCTGATAAATGCTTCAATAATATTGAAAAAGGAAGAGTATGAGTATTCA
ACATTTCCGTGTCGCCCTTATTCCCTTTTTTGCGGCATTTTGCCTTCCTGTTTTTGCTCACCCAGAAACG
CTGGTGAAAGTAAAAGATGCTGAAGATCAGTTGGGTGCACGAGTGGGTTACATCGAACTGGATCTCAACA
GCGGTAAGATCCTTGAGAGTTTTCGCCCCGAAGAACGTTTTCCAATGATGAGCACTTTTAAAGTTCTGCT
ATGTGGCGCGGTATTATCCCGTATTGACGCCGGGCAAGAGCAACTCGGTCGCCGCATACACTATTCTCAG
AATGACTTGGTTGAGTACTCACCAGTCACAGAAAAGCATCTTACGGATGGCATGACAGTAAGAGAATTAT
GCAGTGCTGCCATAACCATGAGTGATAACACTGCGGCCAACTTACTTCTGACAACGATCGGAGGACCGAA
GGAGCTAACCGCTTTTTTGCACAACATGGGGGATCATGTAACTCGCCTTGATCGTTGGGAACCGGAGCTG

AATGAAGCCATACCAAACGACGAGCGTGACACCACGATGCCTGTAGCAATGGCAACAACGTTGCGCAAAC
TATTAACTGGCGAACTACTTACTCTAGCTTCCCGGCAACAATTAATAGACTGGATGGAGGCGGATAAAGT
TGCAGGACCACTTCTGCGCTCGGCCCTTCCGGCTGGCTGGTTTATTGCTGATAAATCTGGAGCCGGTGAG
CGTGGGTCTCGCGGTATCATTGCAGCACTGGGGCCAGATGGTAAGCCCTCCCGTATCGTAGTTATCTACA
CGACGGGGAGTCAGGCAACTATGGATGAACGAAATAGACAGATCGCTGAGATAGGTGCCTCACTGATTAA
GCATTGGTAACTGTCAGACCAAGTTTACTCATATATACTTTAGATTGATTTAAAACTTCATTTTTAATTT
AAAAGGATCTAGGTGAAGATCCTTTTTGATAATCTCATGACCAAAATCCCTTAACGTGAGTTTTCGTTCC
ACTGAGCGTCAGACCCCGTAGAAAAGATCAAAGGATCTTC

Figure S2: CPSA intermediates are not detected in *E. coli* expressing an empty vector control. Lysates of *E. coli* expressing an empty vector control (PQE-80L) were evaluated with LC-MS and SIM for all BPP-linked CPSA intermediates. The Inset depicts a zoomed in region of overlaid chromatograms to resolve less abundant ions.

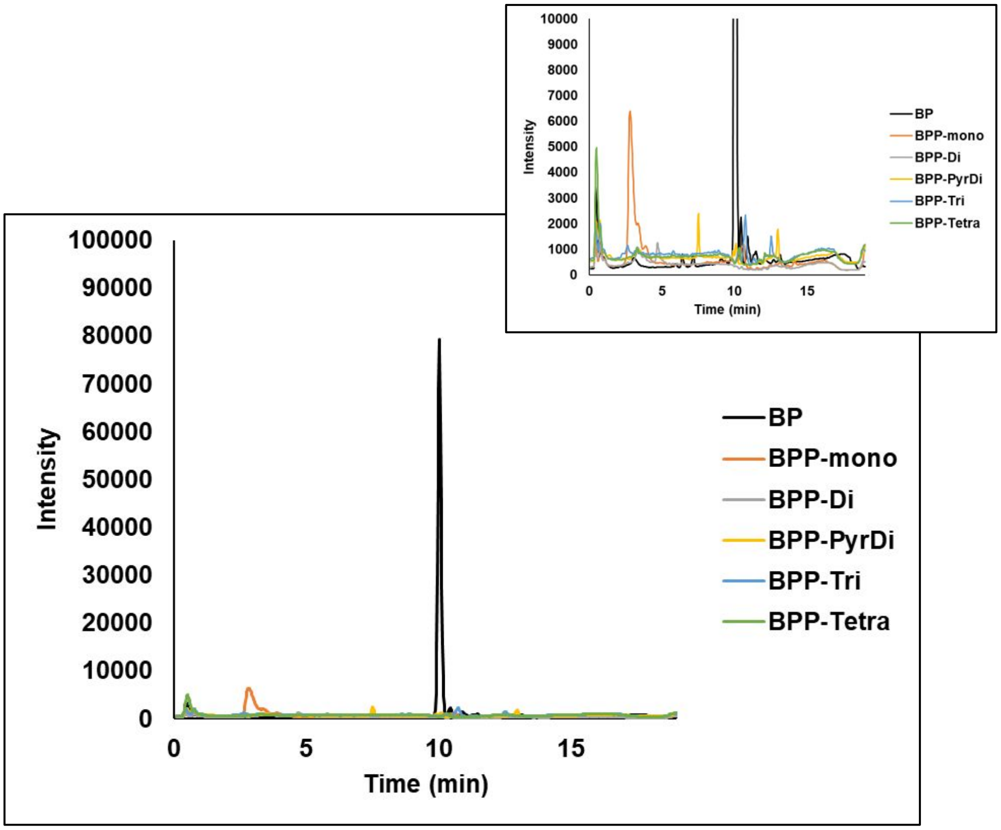

Table S1: Primers used in this study.

Plasmid	Genes	Organism/ template	Primers
pBAS1	*$pglF_{Cj}$*	*C. jejuni ATCC*	5'CATCACCATCACCATCACGACATGATTTTTATAAAAGCAAAAGATTAGC<br>5'GGATTTGTTGGTATTTGGTCATATGTATATCCTCCTTTATACACCTTCTTTATTGTGTTTAAATTC
	$wbpP_{Vv}$	*V. vulnificus* M06-24	5'ATGACCAAATACCAACAAATCC<br>5'GTTATTCTTTATTGATTGTCCCATATGTATATCCTCCTCTATTTTATAAATCGCACATACC
	CPSA operon	*B. fragilis ATCC 9343*	5'ATGGGACAATCAATAAAGAATAAC<br>5'GAGTCCAAGCTCAGCTAATTACTAACGAATGATGCTCCAAAATG
pBAS8	*$pglF_{Cj}$*	*C. jejuni ATCC*	5'GGATCGCATCACCATCACCATCACGgaATGATTTTTATAAAAGCAAAAGATTAGC<br>5'GGTATTTTCATATGTATATCCTCCTTTATACACCTTCTTTATTGTG
	$wcfR_{Bf}$	*B. fragilis ATCC 9343*	5'AGGAGGATATACATATGAAAATACCTTTTTCACCACC<br>5'CAAATGTATATCCTCCTCTAGTGGTGGTGGTGGTGGTGTCGGTTTTCTGCAATTACG
	$wcfS_{Bf}$	*B. fragilis ATCC 9343*	5'CCACTAGAGGAGGATATACATTTGATCCGTTTTTTTGATATCG<br>5'GAGTCCAAGCTCAGCTAATTAGAGCTCCTAACGAATGATGCTCC
**Plasmids below were constructed using SacI digested pBAS8 as vector backbone. See Materials and Methods.**			
pBAS9	*$wcfQ_{Bf}$*	*B. fragilis ATCC 9343*	5'GCATCATTCGTTAGGAGCTCAGGAGGATATACATATGAAATTGGCTGTCATTTC<br>5'CAAGCTCAGCTAATTAGAGCTTCACTTGTCGTCATCGTCTTTGTAGTCACGTACTGTTTTATAGATTATG
pBAS10	*$wcfQ_{Bf}$*	*B. fragilis* ATCC 9343	5'GCATCATTCGTTAGGAGCTCAGGAGGATATACATATGAAATTGGCTGTCATTTC<br>5'CCTTCAACGTACTGTTTTATAGATT
	$wcfO_{Bf}$	*B. fragilis* ATCC 9343	5'AATCTATAAAACAGTACGTTGAAGGAGGATATACATATGAGGAAGATATTATTAACATATGG<br>5'CAAGCTCAGCTAATTAGAGCTTCACTTGTCGTCATCGTCTTTGTAGTCTGACATCATAAATTTATTACATATATTAATTAATC
pBAS11	*$wcfQ_{Bf}$*	*B. fragilis* ATCC 9343	5'GCATCATTCGTTAGGAGCTCAGGAGGATATACATATGAAATTGGCTGTCATTTC<br>5'CCTTCAACGTACTGTTTTATAGATT
	$wcfO_{Bf}$	*B. fragilis* ATCC 9343	5'AATCTATAAAACAGTACGTTGAAGGAGGATATACATATGAGGAAGATATTATTAACATATGG<br>5'CTCATATGTATATCCTCCTTCATGACATCATAAATTTATTACATATATTAATTAATC
	$wcfP_{Bf}$	*B. fragilis* ATCC 9343	5'CATGAAGGAGGATATACATATGAGGGATGGAAAGCC<br>5'CAAGCTCAGCTAATTAGAGCTTCACTTGTCGTCATCGTCTTTGTAGTCTTTATGTAAAAGATTTAAATATATTCTTTC

pBAS12	*$wcfQ_{Bf}$*	*B. fragilis* ATCC 9343	5’GCATCATTCGTTAGGAGCTCAGGAGGATATACATATGAAATTGGCTGTCATTTC<br>5’CCTTCAACGTACTGTTTTATAGATT
	$wcfO_{Bf}$	*B. fragilis* ATCC 9343	5’AATCTATAAAACAGTACGTTGAAGGAGGATATACATATGAGGAAGATATTATTAACATATGG<br>5’CTCATATGTATATCCTCCTTCATGACATCATAAATTTATTACATATATTAATTAATC
	$wcfP_{Bf}$	*B. fragilis* ATCC 9343	5’CATGAAGGAGGATATACATATGAGGGATGGAAAGCC<br>5’CCTCCTTCATTTATGTAAAAGATTTAAATATATTCTTTCGAG
	$wcfM_{Bf}$	*B. fragilis* ATCC 9343	5’AAATCTTTTACATAAATGAAGGAGGATATACATATGAAAAAAAAATATGACTATCTAATTG<br>5’CAACTACAGCAAATATCTTCATATGTATATCCTCCTTCATAAGTCACTATTTATAACTTTTTCCAC
	$wcfN_{Bf}$	*B. fragilis* ATCC 9343	5’ATGAAGATATTTGCTGTAGTTG<br>5’CAAGCTCAGCTAATTAGAGCTTTACTTGTCGTCATCGTCTTTGTAGTCATAGGTAGCTCCATTTTTTTTACG
pBAS15	*$wcfQ_{Bf}$*	*B. fragilis* ATCC 9343	5’GCATCATTCGTTAGGAGCTCAGGAGGATATACATATGAAATTGGCTGTCATTTC<br>5’CCTTCAACGTACTGTTTTATAGATT
	$wcfO_{Bf}$	*B. fragilis* ATCC 9343	5’AATCTATAAAACAGTACGTTGAAGGAGGATATACATATGAGGAAGATATTATTAACATATGG<br>5’CTCATATGTATATCCTCCTTCATGACATCATAAATTTATTACATATATTAATTAATC
	$wcfP_{Bf}$	*B. fragilis* ATCC 9343	5’CATGAAGGAGGATATACATATGAGGGATGGAAAGCC<br>5’GTAAAAGATTTAAATATATTCTTTCGAG
	$wbpP_{Vv}$	*V. vulnificus* M06-24	5’CTCGAAAGAATATATTTAAATCTTTTACATAAATGAAGGAGGATATACATATGACCAAATACGAAAAAATCC<br>5’CAAGCTCAGCTAATTAGAGCTTTATTTTTTATCATTTATAAAGCTTATATACC
pBAS16	*$wcfQO_{Bf}$*	pBAS10	5’GCATCATTCGTTAGGAGCTCAGGAGGATATACATATGAAATTGGCTGTCATTTC<br>5’CTCATATGTATATCCTCCTTCATGACATCATAAATTTATTACATATATTAATTAATC
	$wcfP_{Bf}$	*B. fragilis* ATCC 9343	5’CATGAAGGAGGATATACATATGAGGGATGGAAAGCC<br>5’GTAAAAGATTTAAATATATTCTTTCGAG
	$wbpP_{Vv}$	*V. vulnificus* M06-24	5’CTCGAAAGAATATATTTAAATCTTTTACATAAATGAAGGAGGATATACATATGACCAAATACGAAAAAATCC<br>5’CAAGCTCAGCTAATTAGAGCTTTATTTTTTATCATTTATAAAGCTTATATACC
	$wcfM_{Bf}$	*B. fragilis* ATCC 9343	5’GCTTTATAAATGATAAAAAATAAAGGAGGATATACATATGAAAAAAAAATATGACTATCTAATTG<br>5’CAACTACAGCAAATATCTTCATATGTATATCCTCCTTCATAAGTCACTATTTATAACTTTTTCCAC
	$wcfN_{Bf}$	*B. fragilis* ATCC 9343	5’ATGAAGATATTTGCTGTAGTTG<br>5’CAAGCTCAGCTAATTAGAGCTTTAATAGGTAGCTCCATTTTTTTTACG

pBAS17	*wcfQOP _wbpP_ wcfMN*	pBAS16	5'GCATCATTCGTTAGGAGCTCAGGAGGATATACATATGAA ATTGGCTGTCATTTC<br>5'CCTCCTTTAATAGGTAGCTCCATTTTTTTTACG
	wxz$_{Bf}$	*B. fragilis* ATCC 9343	5'TGGAGCTACCTATTAAAGGAGGATATACATATGGGACAAT CAATAAAGAATAAC<br>5'CCTCCTTCATTGTTTAAATATTGAAAGTAATAATTTC
	wzy$_{Bf}$	*B. fragilis* ATCC 9343	5'CAATATTTAAACAATGAAGGAGGATATACATATGACTAGT ACTTCTTTCTTTATTATTAAG<br>5'AAGCTCAGCTAATTAGAGCTTTAAAATTTGATTTTGGCATT AAAC
pBAS18	*wcfQOP _wbpP_ wcfMN*	pBAS16	5'GCATCATTCGTTAGGAGCTCAGGAGGATATACATATGAA ATTGGCTGTCATTTC<br>5'CCTCCTTTAATAGGTAGCTCCATTTTTTTTACG
	wzx$_{Bf}$	*B. fragilis* ATCC 9343	5'TGGAGCTACCTATTAAAGGAGGATATACATATGGGACAAT CAATAAAGAATAAC<br>5'CAAGCTCAGCTAATTAGAGCTTCATTGTTTAAATATTGAA AGTAATAATTTC
pBAS19	*wcfQOP _wbpP_ wcfMN*	pBAS16	5'GCATCATTCGTTAGGAGCTCAGGAGGATATACATATGAA ATTGGCTGTCATTTC<br>5'CCTCCTTTAATAGGTAGCTCCATTTTTTTTACG
	wzx$_{Bf}$	*B. fragilis* ATCC 9343	5'TGGAGCTACCTATTAAAGGAGGATATACATATGGGACAAT CAATAAAGAATAAC<br>5'CAAGCTCAGCTAATTAGAGCTTCATTGTTTAAATATTGAA AGTAATAATTTC

Table S2: *E. coli* strains used in this study.

Strain	Parent Strain	Genotype	Reference or Source
DH5α		F– φ80lacZΔM15 Δ(lacZYA-argF)U169 recA1 endA1 hsdR17(rK–, mK+) phoA supE44 λ–thi-1 gyrA96 relA1	
MG1655		K-12 F^{-} λ^{-} *ilvG*⁻ *rfb-50 rph-1*	
DR35	MG1655	MG1655 Δwza::kan	DOI: 10.1128/JB.01034-15
BAS5	MG1655	MG1655 ΔwzxB::frt	This study
BAS13	DR35	MG1655 Δwza::frt	This study
MAJ975	MG1655	MG1655 ΔwaaL::frt	This study
BAS24	BAS13	MG1655 Δwza::frtΔwaaL::cam	This study

Table S3: Primers used for *E. coli* mutants.

Strain Designation	Primers	Template	Check Primers
BAS5	5'GAAAGGCTCTTTACGTTAGATGAGC TTATCAGATTAAAATTAATTGCATGAC ATTACACGTCTTGAGCGAT 5'GCACAAACGGCACCAAACAAACCA GAACCAACAATGATATAATCGTACAT ATGAATATCCTCCTTAGTTCC	pKD4	5'TGGCTGCTATTG GGCGAA 5'TCCACCGATATG ATTTCTTTTC
BAS24	5'TCAACAGTCAAGCAGTTTTGGAAAA GTTATCATCATTATAAAGGTAAAACTG AATATCCTCCTTAGTTCC 5'TTGTATAGATAAGAAGTGAGTTTTAA CTCACTTCTTAAACTTGTTTATTCATT GTGTAGGCTGGAGC	pKD3	5'TATCCCAATGGC ATCG 5'ACCCTAATTCAC GTACTCC
MAJ975	5'AGCAGTTTTGGAAAAGTTATCATCA TTATAAAGGTAAAACATTCCGGGGAT CCGTCGACC 5'TAACTCACTTCTTAAACTTGTTTATT CTTAATTAATTGTATGTAGGCTGGAG CTGCTTCG	pKD13	
